# Discovery of Seven ROS-Sensitive Immune Checkpoints and 46 Ligands Mediating Immune Suppression Through T cell-APC Networks

**DOI:** 10.7150/jca.128083

**Published:** 2026-01-14

**Authors:** Baosheng Han, Keman Xu, Fatma Saaoud, Yanjuan Hou, Ying Shao, Yifan Lu, Xiaohua Jiang, Huaqing Zhao, Hong Wang, Xiaofeng Yang

**Affiliations:** 1 Lemole Center for Integrated Lymphatics and Vascular Research, Lewis Katz School of Medicine at Temple University, Philadelphia, PA, 19140, U.S.A.; 2 Metabolic Disease Research; &Thrombosis Research; Department of Cardiovascular Sciences, Lewis Katz School of Medicine at Temple University, Philadelphia, PA, 19140, U.S.A.; 3 Department of Biomedical Education and Data Sciences, Lewis Katz School of Medicine at Temple University, Philadelphia, PA, 19140, U.S.A.; 4 Perelman School of Medicine, University of Pennsylvania, 3400 Civic Center Blvd, Philadelphia, PA 19104, U.S.A.

**Keywords:** CD4^+^Foxp3^+^ regulatory T cells (Treg), immune checkpoints (ICs) and their ligands, antigen presenting cells (APCs), tumor cells, inflammatory cells

## Abstract

**Rationale:** The functional landscape of immune checkpoints (ICs) operating on CD4⁺FoxP3⁺ regulatory T cells (Tregs) remain incompletely defined. Although canonical IC pathways are well characterized, the full spectrum of IC molecules governing Treg-mediated immune regulation across physiological and pathological contexts has not been fully explored.

**Methods:** We performed a comprehensive, multi-dataset transcriptomic screening of Treg membrane proteins to identify candidate immune checkpoints. This approach yielded 151 putative novel ICs, including 45 Treg-specific molecules and 106 FoxP3⁺-upregulated candidates. Cross-referencing these candidates with ten well-established IC-deficient models refined the list to 85 high-confidence ICs. A subsequent high-stringency, integrating expression specificity, functional relevance, and cross-dataset consistency, was applied to identify seven the most robust candidates. Ligand-receptor interaction mapping was then performed to define associated IC ligands and characterize their cellular expression patterns.

**Results:** This integrative analysis identified seven previously unrecognized immune checkpoints: CEP55, CD38, EHD4, CD200R1, PRC1, RAPH1, and CD86 expressed across Tregs and multiple T cell subsets. Ligand interaction mapping further revealed 46 corresponding IC ligands, predominantly expressed on antigen-presenting cells and tumor cells. Together, these IC-ligand interactions form extensive regulatory networks that modulate immune signaling and inflammatory responses.

**Conclusion:** Our study delineates a comprehensive immune checkpoint-ligand network encompassing seven novel ICs and 46 associated ligands, providing mechanistic insight into Treg- and T cell-mediated immune regulation. This expanded IC landscape broadens the current repertoire of immune modulatory pathways and highlights new therapeutic opportunities across cancer, autoimmune disorders, infectious diseases, transplantation immunology, inflammatory conditions, and cardiovascular diseases.

## Introduction

Upon stimulation by pathogen/danger-associated molecular patterns (PAMPs/DAMPs) 1, both innate and adaptive immune responses 2-13 are activated through diverse cellular mediators. This includes antigen-presenting cells (APCs), such as monocytes, macrophages, dendritic cells (DCs), B cells 14, 15, innate immune endothelial cells 16-18, CD4⁺FoxP3⁺ regulatory T cells (Tregs) 19-24, and trained immune cells 25. Soluble factors, including cytokines, secretomes 26-28, and exosomes, along with membrane-bound co-signaling 29 and immune checkpoint (IC) receptors 29, 30, further shape the immune response landscape by governing inter-cellular interaction in immunopathological contexts.

CD4⁺FoxP3⁺ Tregs are key immunosuppressive cells that suppress immune responses to PAMPs/DAMPs and various antigens 31, thereby maintaining peripheral tolerance and preventing autoimmune and pathological immune reactions. The significance of Tregs in inhibiting the pathogenesis of diseases is further highlighted by 2025 Nobel Prize for identification of Tregs 32. Although Tregs were initially characterized by the expression of CD25 (IL2Rα), they are now more precisely defined by the lineage-specifying transcription factor Forkhead Box P3 (FoxP3) 33, which is essential for their immunosuppressive function 34-36. We recently proposed that pathological conditions can reprogram physiological Tregs into pathological Tregs with reduced immunosuppressive function and increased plasticity 24, 31, 37, 38, through both antigen-dependent and -independent mechanisms. Our studies highlight Treg cell death pathways, IL-35 production, and epigenetic regulation as potential therapeutic targets to preserve Treg stability 22, prevent the transition to pathological, plastic, or antigen-presenting Tregs 39, and suppress inflammation 40 via their secretomes, including exosomes 41, 42. It is well recognized that Tregs can acquire phenotypic and functional features of other T helper subsets under disease conditions 28, 41, 43-45, a process influenced by co-inhibitory/immune checkpoint receptors 29. However, beyond the known checkpoints, it remains unclear whether other Treg membrane proteins may function as novel immune checkpoints that modulate their suppressive activity.

Immune checkpoint therapy (ICT) has revolutionized cancer treatment by blocking Treg-mediated immunosuppression 34, enhancing anti-tumor T cell responses 3-5, 19, 46, and significantly improving clinical outcomes, including durable remission or cure in a subset of patients 47, 48. Despite these advances, ICT monotherapy response rates remain modest (~20%) 48, suggesting the involvement of multiple ICs in sustaining Treg-mediated suppression and T cell exhaustion. Currently, at least 22 ICs are known to be expressed on Tregs, where they regulate Treg functions and serve as lineage markers. These ICs interact with other membrane receptors and transcription factors to define 67 distinct Treg subpopulations linked to autoimmunity, transplantation, and tumor immunity 49. However, a significant knowledge gap remains: beyond these known checkpoints, how many other Treg-expressed membrane proteins function as new inhibitory ICs? Addressing these questions is critical for expanding the new therapeutic landscape of immune modulation in cancer and chronic inflammatory diseases.

## Materials and Methods

### Generation of cell type-specific gene lists and collection of transcriptomic resources

To comprehensively investigate IC regulation across immune cell types, we curated gene expression datasets from two primary sources: the Human Protein Atlas (HPA; https://www.proteinatlas.org/) and the NIH NCBI Gene Expression Omnibus (GEO; https://www.ncbi.nlm.nih.gov/gds). From the HPA, we extracted cell type-specific gene signatures for major immune cell populations, including CD4⁺FoxP3⁺ Tregs, monocytes, B cells, macrophages, and dendritic cells. This database provides protein-coding gene expression data based on transcriptomics and antibody-based proteomics, offering one of the most comprehensive resources for human cell-specific protein expression. Specifically, we retrieved 2,202 annotated plasma membrane proteins, 431 Treg-specific proteins, 503 macrophage-related proteins, 16,406 B cell-related proteins, 481 dendritic cell-related proteins, and 225 monocyte-related proteins. These gene sets were used to define and refine our lists of candidates IC proteins and ligands with potential cell type-specific immunoregulatory functions.

In parallel, a wide range of immune- and IC- relevant transcriptomic datasets with gene deficiencies were collected from the GEO database. These included: IC deficiency datasets (e.g., CTLA-4, PD-1, LAG3, TIGIT knockouts or knockdowns); antibody blockade datasets involving inhibitory ICs; datasets of tumor-infiltrating T cells and Tregs; T cell activation datasets; datasets involving loss-of-function models of reactive oxygen species (ROS) regulators, including NFE2 like BZIP transcription factor 2 (NRF2) and related antioxidant transcription factors. These datasets were used in a three-step screening pipeline to identify novel ICs and their ligands based on expression dynamics in both physiological and pathological immune contexts. All dataset accession numbers (GEO IDs) were provided in the corresponding figure legends, and detailed comparisons, analysis criteria, and results were presented in the supplementary figures.

### Characterizing IC expression across immune cell subsets using single-cell RNA sequencing (scRNA-seq)

To investigate the expression profiles of newly identified ICs at single-cell resolution, we analyzed scRNA-Seq data from the MIT Single Cell Portal (SCP1186). This dataset originates from a study published in Cell 50, which profiled cellular populations within lymph nodes, including immune cells and sensory neurons with immunomodulatory potential. We specifically explored the expression patterns of candidate IC genes across diverse immune cell subsets, including T cells, B cells, macrophages, and dendritic cells, to assess their potential regulatory roles in lymphoid tissue microenvironments.

### Mapping antigen-presenting cell subsets associated with novel ICs

To determine the antigen-presenting cell types most likely to interact functionally with each newly identified IC, we first queried each IC gene in the NCBI Gene database. For each checkpoint, we extracted the full list of interacting genes from the “Interactions” section, representing potential binding partners or regulatory targets that have been experimentally identified. These interaction gene sets were then systematically cross-referenced with a curated database of plasma membrane proteins to prioritize surface-expressed candidates likely to mediate cell-cell communication. Next, to map these interaction partners to specific APC types, we overlapped the filtered gene lists with cell type-specific expression signatures of four key APC populations: macrophages, monocytes, B cells, and dendritic cells. This integrative approach allowed us to determine the specific APC populations most likely to serve as functional cellular partners of each novel IC. For example, we identified 124 interaction proteins for human CTLA4 (https://www.ncbi.nlm.nih.gov/gene/1493), 8 interaction proteins for human KLRG1 (https://www.ncbi.nlm.nih.gov/gene/10219), 16 interaction proteins for human LAG3 (https://www.ncbi.nlm.nih.gov/gene/3902), 84 interaction proteins for human PD-1 (https://www.ncbi.nlm.nih.gov/gene/5133), and 25 interaction proteins for human TIGIT (https://www.ncbi.nlm.nih.gov/gene/201633).

### Statistical methods

All data analyses, particularly the correlation analyses between known and newly identified ICs were conducted in consultation with a biostatistics expert among the co-authors to ensure the use of appropriate statistical methods and accurate interpretation. Gene expression data presented in the manuscript were derived from GEO datasets, with differential expressions determined using a significance threshold of *p* < 0.05. Fold changes were reported as log₂-fold change (log₂FC) to facilitate interpretation of gene regulation patterns.

## Results

### 1. Identification of 151 novel inhibitory immune checkpoint candidates, including 45 Treg-specific and 106 FoxP3-upregulated membrane proteins

**Figure 1:**
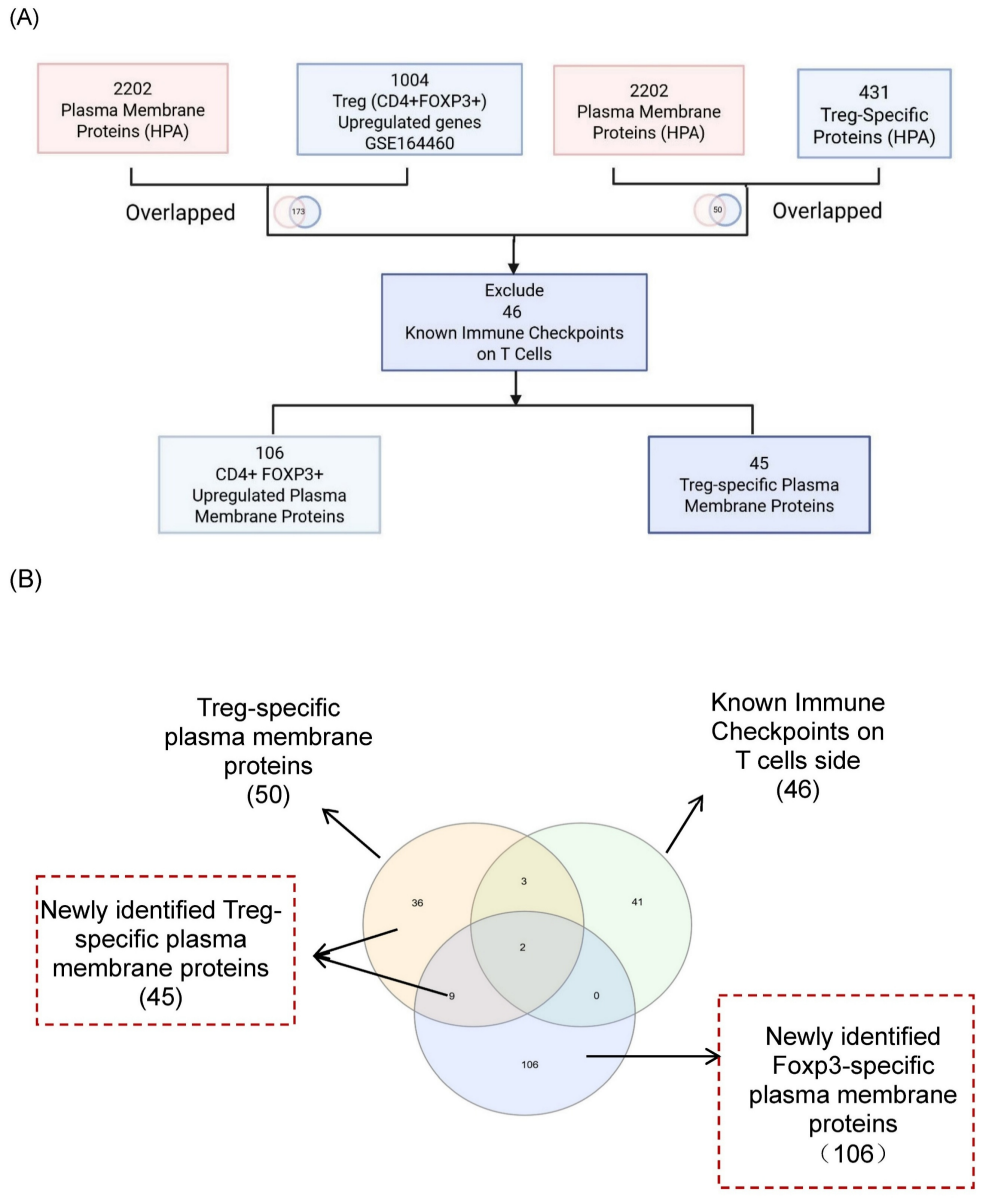

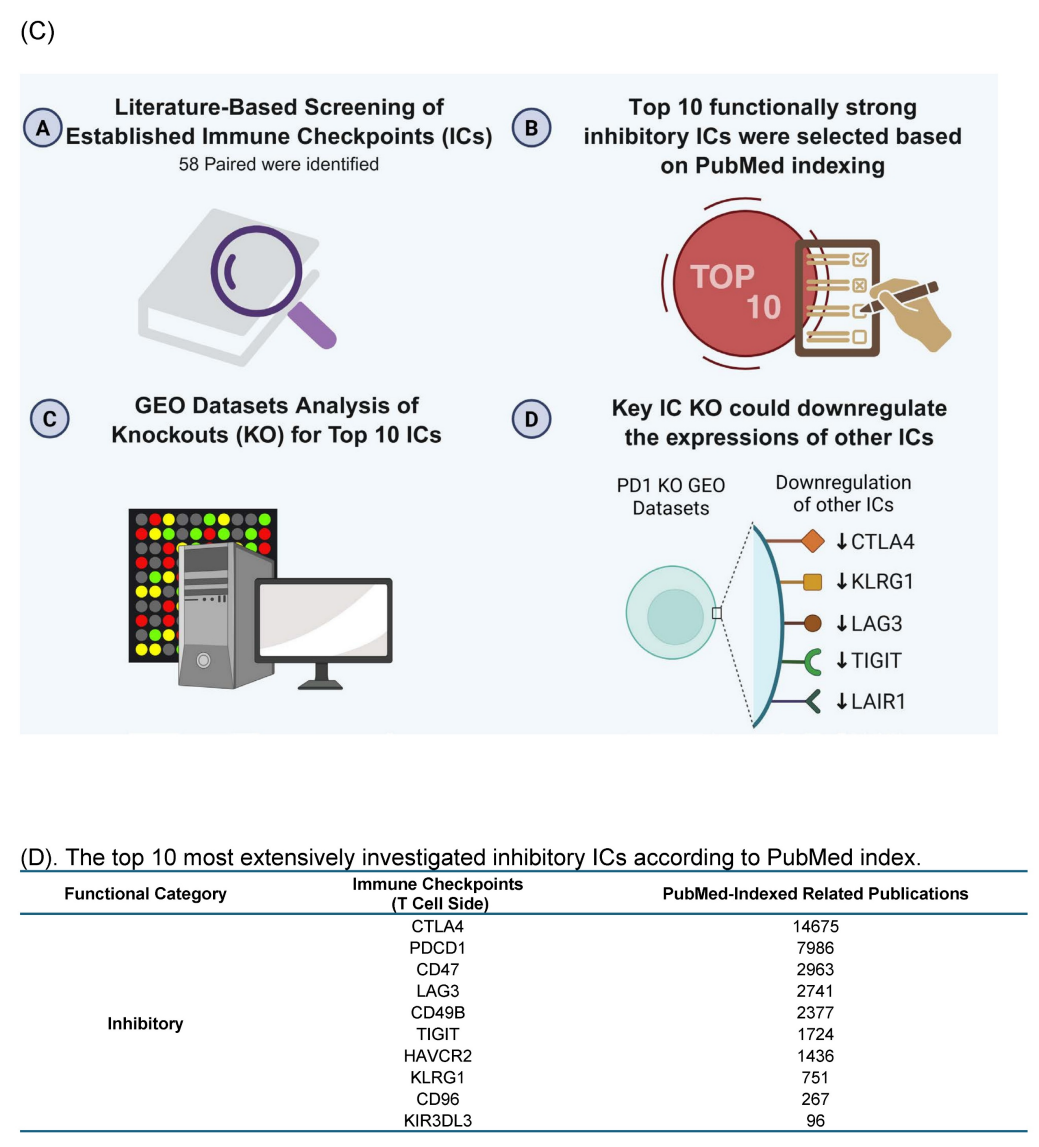

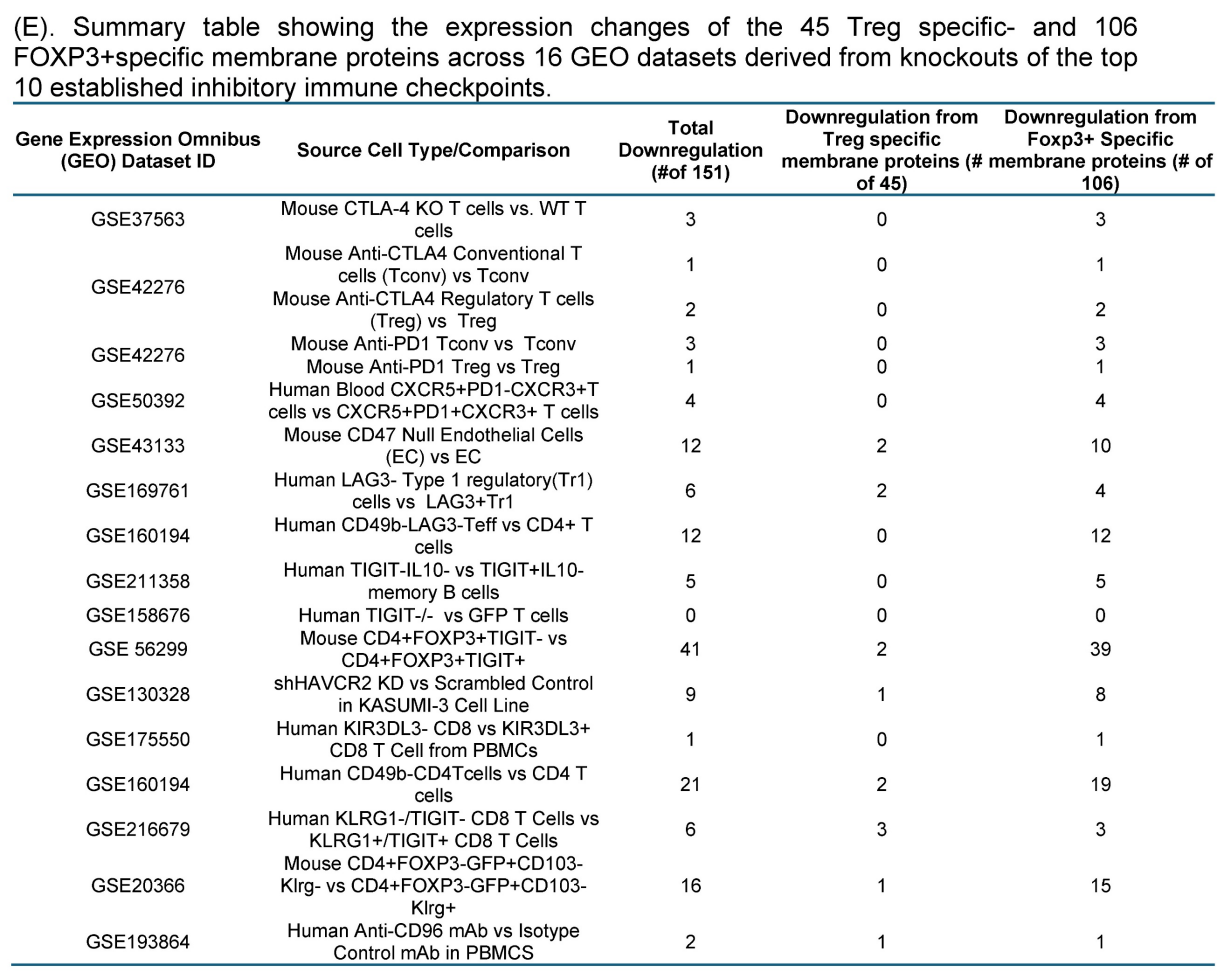
The Workflow for Identifying a Gene List for Potential Immune Checkpoint Candidates. (A) Flowchart illustrates the multi-step strategy used to identify candidate immune checkpoint genes. A total of 2,202 plasma membrane proteins were obtained from the Human Protein Atlas (HPA), and 1,004 significantly upregulated genes in CD4⁺FOXP3⁺ regulatory T (Treg) cells were retrieved from the NIH-NCBI Gene Expression Omnibus Datasets GSE164460 RNA microarray dataset. The intersection of these two datasets yielded 173 overlapping genes. On the other hand, 431 Treg-specific genes from the HPA overlapped with the same set of 2,202 plasma membrane proteins, resulting in 50 shared genes. (B) After excluding 46 known immune checkpoints expressed on the T-cell side, 106 CD4⁺FOXP3⁺ upregulated plasma membrane proteins and 45 Treg-specific plasma membrane proteins were identified. The complete gene lists were provided in Supplementary Table 1. (C) Research strategy for identification of immune checkpoint clusters. We performed literature screening to compile 58 established immune checkpoints (ICs). From these, the top 10 most frequently studied inhibitory or stimulatory ICs indexed in PubMed were selected as primary knockout targets. Experimental knockouts (GEO Datasets) of these targets were then analyzed to determine whether the remaining established ICs exhibited similar expression trends, confirming the presence of IC clusters. (D-E) The tables illustrate how well-characterized immune checkpoints were leveraged to uncover novel immunosuppressive IC candidates. * The table details could be found in Supplementary Table.

It has been reported that as many as 22 ICs have been used in classifying 67 Treg subpopulations 49, suggesting the significance of ICs in Treg immunosuppressive functions. Therefore, we hypothesized that Treg plasma membrane proteins and Treg-specific Foxp3-upregulated plasma membrane proteins are ideal candidates for screening for new ICs. As outlined in Fig. 1A, B, and C, we developed a multi-step strategy to identify new IC candidates based on dramatic progress in determination of cell-type specific protein genes. A total of 2,202 plasma membrane proteins were obtained from the HPA, and 1,004 significantly upregulated genes in CD4⁺FOXP3⁺ Treg were retrieved from the NIH-NCBI Gene Expression Omnibus Datasets GSE164460 RNA microarray dataset 51. The Venn Diagram was used to intersect these two datasets and yielded 173 overlapping genes, indicating that 173 genes encoded for Foxp3-upregulated plasma membrane proteins. On the other hand, 431 Treg-specific genes from the HPA also overlapped with the same set of 2,202 plasma membrane proteins, resulting in identification of 50 human Treg plasma membrane protein genes. After excluding 46 known IC receptors expressed on the T-cell membrane, 106 FoxP3⁺upregulated plasma membrane proteins and 45 Treg-specific plasma membrane proteins were identified as the candidates for new inhibitory ICs.

### 2. Functional screening of known inhibitory immune checkpoint deficiencies identifies downregulated Treg- and FoxP3⁺-associated IC candidates

We performed literature screening to compile 58 reported ICs 30. Based on the publication numbers on the PubMed database, the top 10 most frequently studied inhibitory ICs including CTLA4, PDCD-1, CD47, LAG3, CD49B, TIGIT, HAVCR2, KLRG1, CD96, and KIR3DL3 were selected, and their corresponding IC knockout (KO) datasets were collected from the NIH-NCBI GeoDatasets database as the primary known inhibitory ICs with strong immunosuppressive functions (Fig. 1D).

A significant paper published on Cancer Cell reported that combined anti-PD-1 and CTLA4 therapy induces higher CD8 T cell infiltration into tumor than anti-PD-1 monotherapy 52, suggesting that two inhibitory ICs PD-1 and CTLA4 may have different effects on T cell and Treg transcriptomics, and PD-1 and CTLA4 also have functional synergy in suppressing T cell immunity; thus combined anti-PD-1 and CTLA4 therapy induces higher TIGIT (another inhibitory IC) expression than anti-PD-1 monotherapy 52, suggesting that both PD-1 and CTLA4 downregulate TIGIT expression, and inhibitory ICs may not only upregulate other inhibitory ICs for functional clustering/collaboration but also downregulate the expressions of other inhibitory ICs. In addition, a recent single-cell transcriptomic analyses published in Nature communication have revealed that Tregs are highly heterogeneous rather than a uniform immunosuppressive population. Only a small core set of genes, including FOXP3 and CTLA4, is consistently expressed across all Tregs, whereas many inhibitory molecules exhibit context-dependent and state-specific expression patterns. Importantly, TCR signaling does not determine Treg lineage commitment but instead shapes distinct activated Treg states, indicating that immunosuppressive functions are dynamically regulated. Furthermore, substantial transcriptional overlap between Tregs and conventional T cells highlights a continuum of immune states rather than a rigid dichotomy. Together, these findings support the concept that inhibitory ICs operate as interconnected functional networks rather than isolated or linearly compensatory pathways 53.

**Figure 2:**
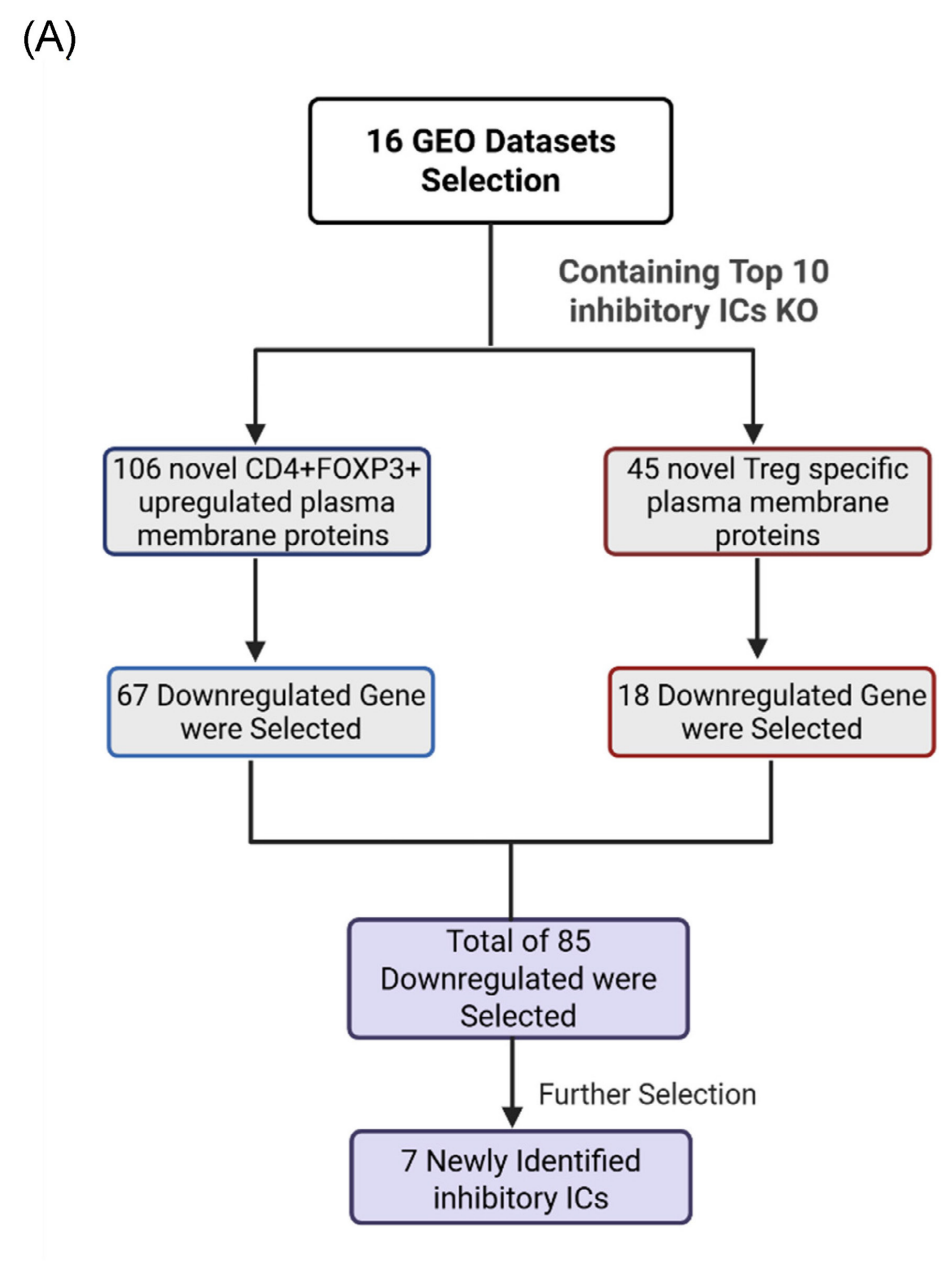

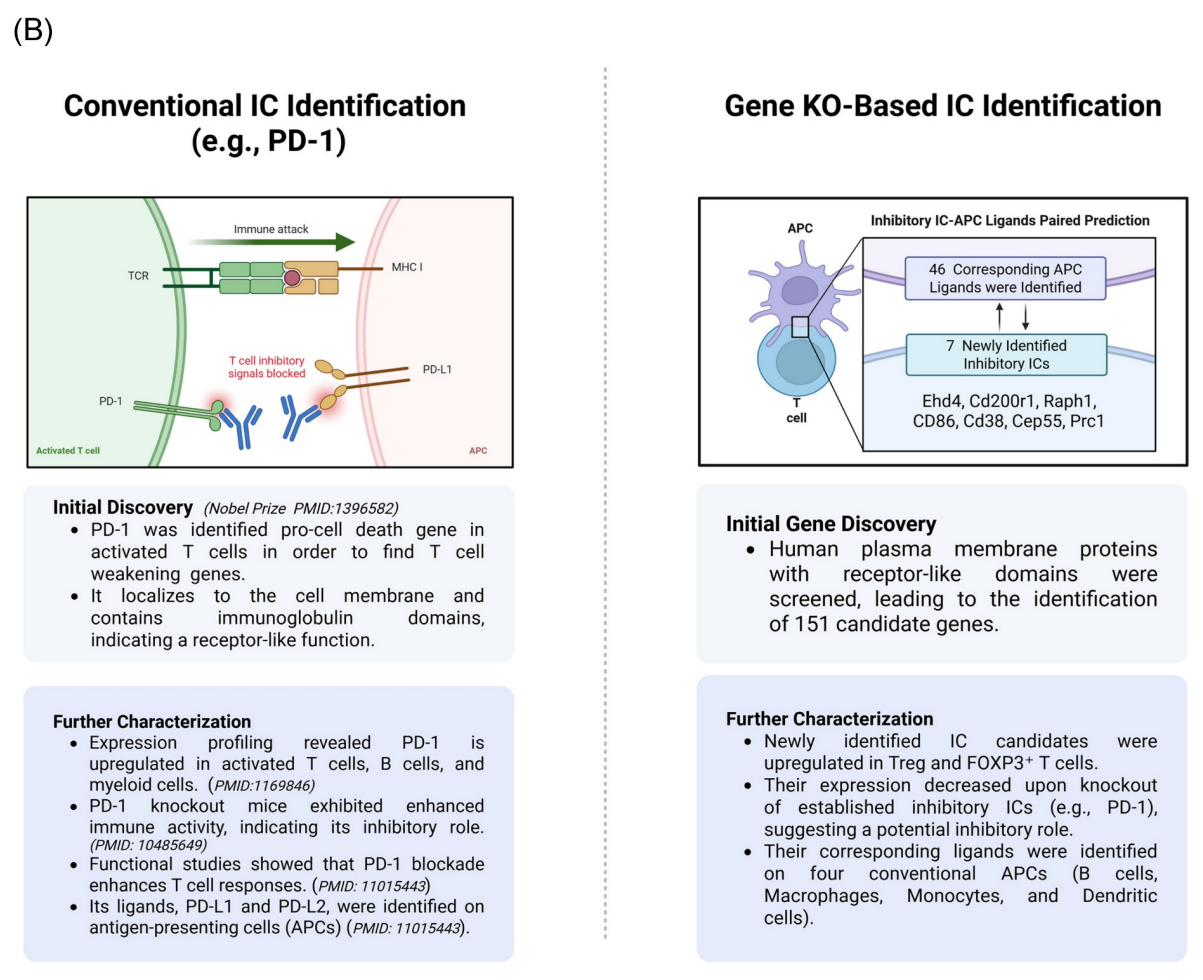
Research Strategy for the Identification of Newly Identified Immune Checkpoints. (A) The flow chart indicates the detailed strategy used to identify novel immune checkpoints with inhibitory function. (B) We applied a comparable strategy to conventional immune checkpoint (IC) identification, using PD-1 as a reference example. PD-1 was initially identified in mice through a screen for apoptosis-related genes and found to localize on the cell membrane, containing immunoglobulin-like domains indicative of a receptor function. Subsequent studies confirmed PD-1 expression on activated T cells, and knockout models demonstrated enhanced T cell responses, supporting its inhibitory role. Inspired by this approach, we first intersected Treg- and FOXP3⁺-specific gene signatures with the human plasma membrane protein database to prioritize surface proteins with receptor-like domains. Next, we screened GEO datasets for genes whose expression was consistently downregulated following the knockout of established inhibitory ICs, suggesting a similar inhibitory function. Finally, we predicted and validated their corresponding APC ligands, enabling the identification of novel inhibitory immune checkpoint pairs.

Consistent with this framework, transcriptomic perturbation approaches such as immune checkpoint blockades or genetic deficiency models have been widely used to infer immunosuppressive function by identifying coordinately regulated gene modules 54. In line with single-cell evidence showing that only a limited core of inhibitory molecules is universally expressed while others are dynamically regulated, we leveraged transcriptomic datasets from established IC-deficient or blockade models to systematically prioritize novel inhibitory IC candidates for further functional characterization. We hypothesized that the expressions of 151 new IC candidates can be downregulated in deficiencies of key inhibitory IC transcriptomic datasets. To test the hypothesis, 16 experimental gene KO transcriptomic datasets were collected from the NIH-NCBI-GEO Datasets database including 3 datasets on each of four inhibitory ICs such as CTLA4, PD-1 and TIGIT, KLRG and other ICs. As shown in Fig. 1E, deficiencies or blockade of five ICs including CD47 (CD47 null endothelial cells versus (vs) CD47^+^ endothelial cells), TIGIT (mouse CD4^+^Foxp3^+^TIGIT^-^ vs CD4^+^Foxp3^+^TIGIT^+^), HAVCR2 (shHAVCR2 knock-down (KD) vs scrambled control in KASUMI-3 human lymphoblast cell), CD49b (CD49b^-^human CD4^+^ T cell vs CD49b^+^CD4^+^ T cell), KLRG (CD4^+^Foxp3^-^GFP^+^CD103^-^Klrg^-^ vs CD4^+^Foxp3^-^GFP^+^CD103^-^Klrg^+^), significantly downregulated 12 (7.9%), 41 (27.2%), 9 (5.9%), 21 (13.9%), and 16 (10.5%) out of 151 new inhibitory IC candidates, respectively. These results demonstrated that deficiencies of known ICs partially downregulate the expressions of new IC candidates in Treg, T cell and other cell types, suggesting that the majority of new IC candidates are immunosuppressive. We then rationed that new IC candidates were qualified for further characterization if the expressions of new IC candidates were downregulated in any of 16 known IC deficient and /or blockade datasets. As shown on Fig. 2A, 18 out of 45 Treg-identified IC candidates and 67 out of 106 Foxp3 upregulated IC candidates were selected in the first IC gene KO functional screening for further characterization based on their expressions were downregulated at least in one of 16 known IC deficient or blockade datasets.

### 3. High-Hierarchy IC Deficiencies Downregulate 20% of Known ICs and Identify 7 Novel Candidates from 151 Original ICs via Third-Round Stringent Screening

We hypothesized that by following the experimental logic historically used to identify classical immune checkpoints (ICs), we could similarly identify new ICs supported by experimental data. As illustrated in the left panel of Fig. 2B, PD-1 serves as a prototypic example of this logic. PD-1 was first recognized as a pro-cell death gene in activated T cells, indicating its role in weakening immune responses. Subsequent studies localized PD-1 to the plasma membrane 55, where it mediates intercellular receptor-ligand interactions. PD-1 is expressed on activated T cells, B cells, and myeloid cells, and its genetic deficiency in mice leads to enhanced immune activation, confirming its immunosuppressive function. Furthermore, its ligands, PD-L1 and PD-L2, are expressed on antigen-presenting cells (APCs), facilitating PD-1-PD-L1-mediated inhibitory signaling between immune cells.

Building on this established framework, we developed a new multi-step strategy to identify novel ICs, as shown in the right panel of Fig. 2B. In the first stage, we screened for plasma membrane proteins potentially involved in immunosuppression by integrating Treg-specific proteins from the Human Protein Atlas (HPA) with FoxP3⁺ Treg-upregulated genes derived from GEO transcriptomic datasets. This approach yielded 151 candidate ICs, including both Treg-specific and FoxP3-upregulated membrane proteins. To further evaluate their immunosuppressive potential, we subjected these candidates to two additional rounds of screening against high-hierarchy classical IC-deficient transcriptomic datasets. Through this iterative filtering process, we aimed to identify IC candidates that share core characteristics with established inhibitory checkpoints such as PD-1.

A fundamental principle in immunogenetics holds that the deficiency of a master inhibitory immune checkpoint (IC) gene, such as PDCD1 (PD-1), disrupts the immunosuppressive functions mediated by that pathway, leading to the downregulation of other inhibitory IC genes functionally associated with PD-1 signaling 56. Based on this concept, we hypothesized that the ten best-characterized ICs could be stratified into high-hierarchy ICs, which occupy upstream positions in regulatory signaling cascades and promote the expression of multiple downstream inhibitory ICs, and low-hierarchy ICs, which influence only a limited subset of other inhibitory checkpoints.

**Figure 3:**
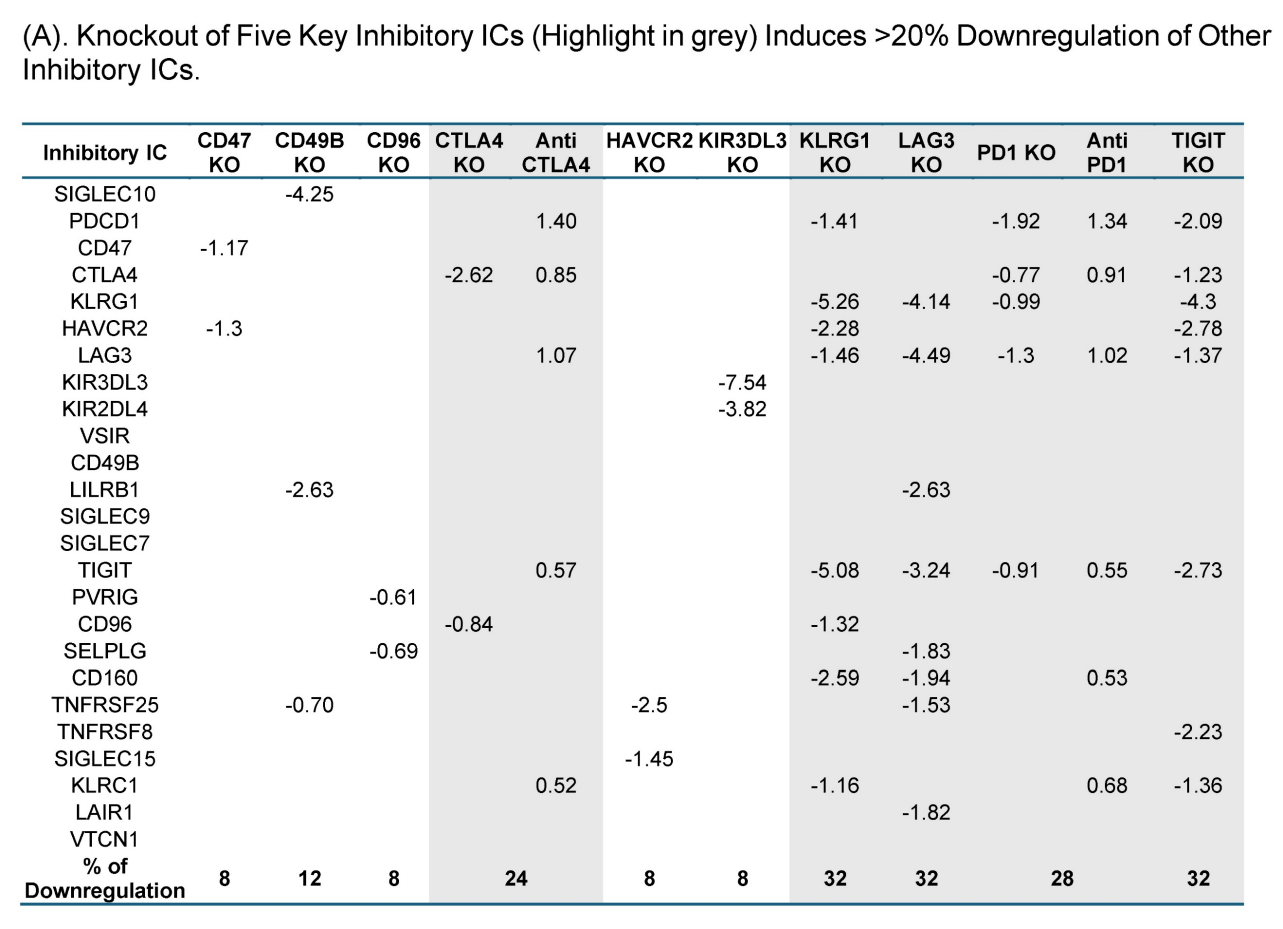

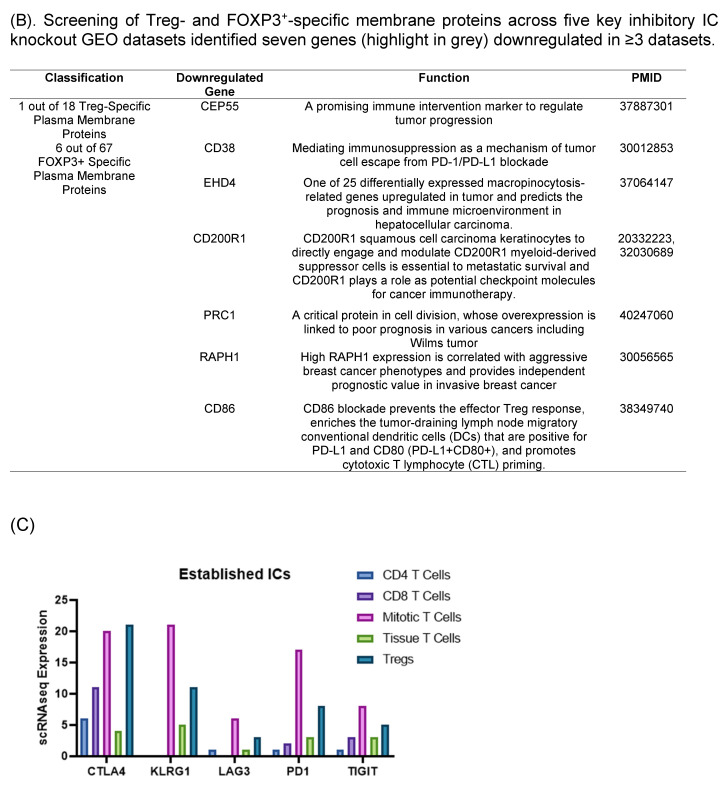

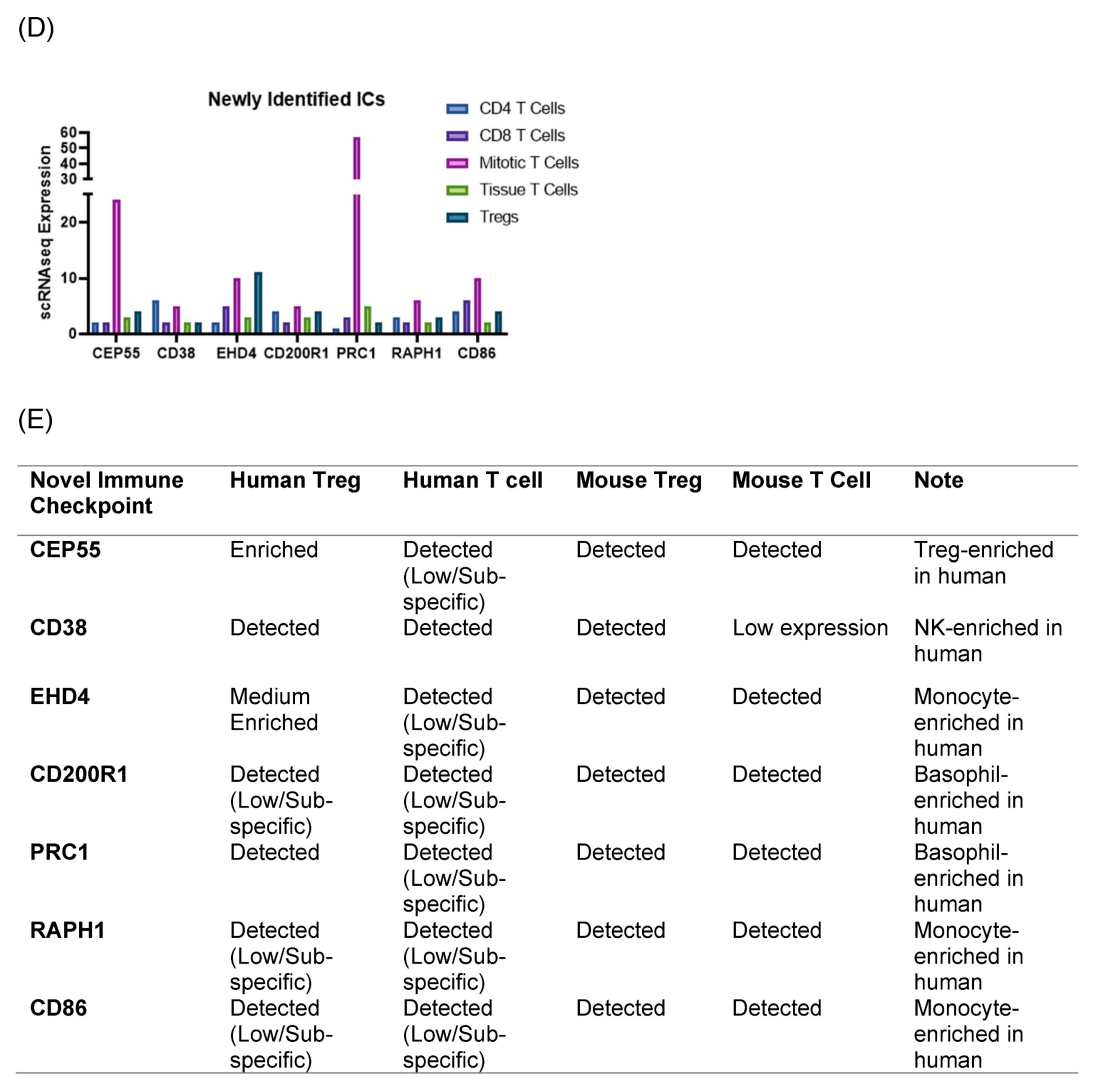
Knockout Screening Validates the Immunosuppressive Roles of Novel Immune Checkpoint Candidates Identified by Their Downregulation in Established Inhibitory IC Knockout Transcriptomic Datasets. (A). We first selected 25 well-established inhibitory immune checkpoints expressed on T cells and screened them across 16 GEO datasets containing knockouts of the top 10 inhibitory immune checkpoints. If the knockout of any of these top 10 checkpoints resulted in a decrease of more than 20% in the expression of other inhibitory checkpoints, indicative of immunosuppressive function. Five checkpoints—CTLA4, KLRG1, LAG3, PD1, and TIGIT—exhibited this key function and were used to refine the criteria for identifying novel inhibitory immune checkpoints. (B). We then screened newly identified 45 Treg- and 106 FOXP3⁺-specific plasma membrane proteins across the GEO knockout datasets of these five checkpoints. Genes that were downregulated at least three out of the five datasets were considered as potential inhibitory candidates. A total of seven such genes were identified (highlighted in grey): Ehd4, Cd200r1, Raph1, Bmpr2, Cd38, Cep55, and Prc1. Of these, the Treg-associated inhibitory group identified CEP55, while the FOXP3⁺ group identified Ehd4, Cd200r1, Raph1, Bmpr2, Cd38, and Prc1. (C). Figure C illustrates the expression patterns of five well-established inhibitory ICs in lymph node T cell subsets using single-cell RNA sequencing (scRNA-seq) data. These ICs including CTLA4, KLRG1, LAG3, PD1, and TIGIT were expressed across CD4⁺ T cells, CD8⁺ T cells, mitotic T cells, tissue-resident T cells, and regulatory T cells (Tregs). Figure D shows comparable expression profiles for seven newly identified inhibitory IC candidates: CEP55, CD38, EHD4, CD200R1, PRC1, RAPH1, and CD86 demonstrating similar distribution across the same T cell subsets. (E) Cross-species expression summary of seven newly identified immune checkpoint receptors in Tregs and conventional T cells.

To test this hypothesis, we examined the expressions of 25 known ICs in 10 best characterized IC deficient datasets. As shown in Fig. 3A, CTLA4 KO, KLRG1 KO, LAG3 KO, PD-1 KO and TIGIT KO downregulated 24%, 32%, 32%, 28%, and 32%, respectively, which were classified into the high hierarchy group. The rest five best characterized ICs including CD47, CD49B, CD96, HAVCR2, and KIR3DL3 were classified into the low hierarchy group. Taken together, our results demonstrated for the first time that the expressions of inhibitory ICs are clustered into two groups with both high and low hierarchies. The gene deficient datasets of the high hierarchy group of inhibitory ICs were selected as screening tools for immunosuppressive functions of new IC candidates.

We hypothesized that to secure the potential immunosuppressive functions of new inhibitory IC candidates with a high confidence, downregulation of new inhibitory IC candidates in ≥3 out of 5 high-hierarchy inhibitory IC deficient transcriptomic datasets including deficiencies of CTLA4, PD1, LAG3, TIGIT and KLRG1 would significantly enhance the likelihood of their immunosuppressive functions. As shown in Fig. 3B, one out of 18 Treg derived inhibitory IC candidates, CEP55, and 6 out of 67 Foxp3-upregulated inhibitory IC candidates including CD38, EHD4, CD200R1, PRC1, RAPH1, and CD86 were selected, respectively, based on their downregulation in ≥ 3 out of 5 high-hierarchy inhibitory IC deficient transcriptomic datasets. These results demonstrated that after the third IC gene KO functional screen, seven out of 151 new IC candidates such as centrosomal protein 55 (CEP55), CD38, EH domain containing 4 (EHD4), CD200R1, protein regulator of cytokinesis 1 (PRC1), Ras association (RalGDS/AF-6) and pleckstrin homology domains 1 (RAPH1), and CD86 were qualified as new ICs with top confidence. Taken together, these reports from other teams with experimental evidence strongly support our finding that these 7 new IC candidates including CEP55, CD38, EHD4, CD200R1, PRC1, RAPH1, and CD86 are new ICs with immune suppressive functions, which have been supported with strong experimental data.

### 4. Single-cell transcriptomic analysis reveals broad T cell and Treg expression of seven newly identified inhibitory immune checkpoints

We hypothesized that 7 new ICs were expressed in T cells and Treg (Figure 3C-D). To examine this hypothesis, we searched for single cell RNA-sequencing (scRNA-Seq) data at MIT-Broad Institute Single Cell Portal database. We identified a scRNA-Seq dataset from lymph nodes 50, where gene expressions at T cell and Treg were characterized, suggesting that the scRNA-Seq data were patho-physiologically relevant. The RNA expressions of 7 new ICs including CEP55, CD38, EHD4, CD200R1, PRC1, RAPH1, and CD86 were found in five T cell populations including CD4^+^ T cells, CD8^+^ T cells, mitotic T cells, tissue T cells and Treg 50. Since all the scRNA-Seq data were from the same dataset and statistically comparable, the RNA expressions of five best characterized ICs such as CTLA4, KLRG1, LAG3, PD1 and TIGIT were also found in CD4^+^ T cells, CD8^+^ T cells, mitotic T cells, tissue T cells and Treg. All the 5 best characterized ICs and 7 new ICs had the highest expression in mitotic T cells except CD38. The expression levels of five best characterized ICs in Treg except LAG3 were above 5, whereas the expression levels of 7 new ICs in Treg were lower than 5 except EHD4. The expression ratio between CD8^+^ T cells versus CD4^+^ T cells in the 5 best characterized ICs were larger than 1 for CTLA4, PD-1 and TIGIT whereas the expression ratio between CD8^+^ T cells versus CD4^+^ T cells in the 7 new ICs were ≥ 1 except CD38, CD200R1, and RAPH1. Taken together, our results using scRNA-Seq data once again demonstrated that 7 new ICs were expressed in CD4^+^ T cells, CD8^+^ T cells, mitotic T cells, tissue T cells and Treg; and the expression patterns of 7 new ICs were slightly different from that of 5 best characterized ICs in T cells and Treg but similar.

To further consolidate the expression patterns of the seven newly identified IC candidates and their corresponding ligands, we generated two complementary summary tables by integrating curated public transcriptomic resources from the Human Protein Atlas (HPA) and ImmGen. The expression of those seven ICs in Tregs and conventional T cells from both human and mouse is summarized in Figure 3E. This table provides a qualitative overview of relative cell-type bias (e.g., enriched, detected, or low/subset-specific), enabling cross-species comparison without reliance on quantitative expression thresholds. Notably, the summarized expression patterns confirm that the newly identified IC receptors and their corresponding ligands are indeed expressed in the expected immune cell compartments, supporting our hypothesis that these candidates participate in immune checkpoint-mediated regulatory networks. In parallel, given the larger number of candidate ligands, the expression patterns of 46 newly identified IC ligands across antigen-presenting cells were compiled in a separate Supplementary Table 1. Human APC expression was curated from HPA immune cell datasets, which primarily include monocytes and dendritic cells derived from peripheral blood, whereas mouse APC expression, including macrophage populations was assessed using Immunological Genome Project (ImmGen) datasets. Together, these tables complement the single-cell RNA-seq analysis shown in Figure 3 by integrating cross-species, multi-dataset expression evidence and providing a structured framework for prioritizing immunosuppressive IC candidates.

### 5. Identification of 46 novel ligands for seven newly discovered inhibitory immune checkpoints in tolerogenic antigen-presenting cells

**Figure 4:**
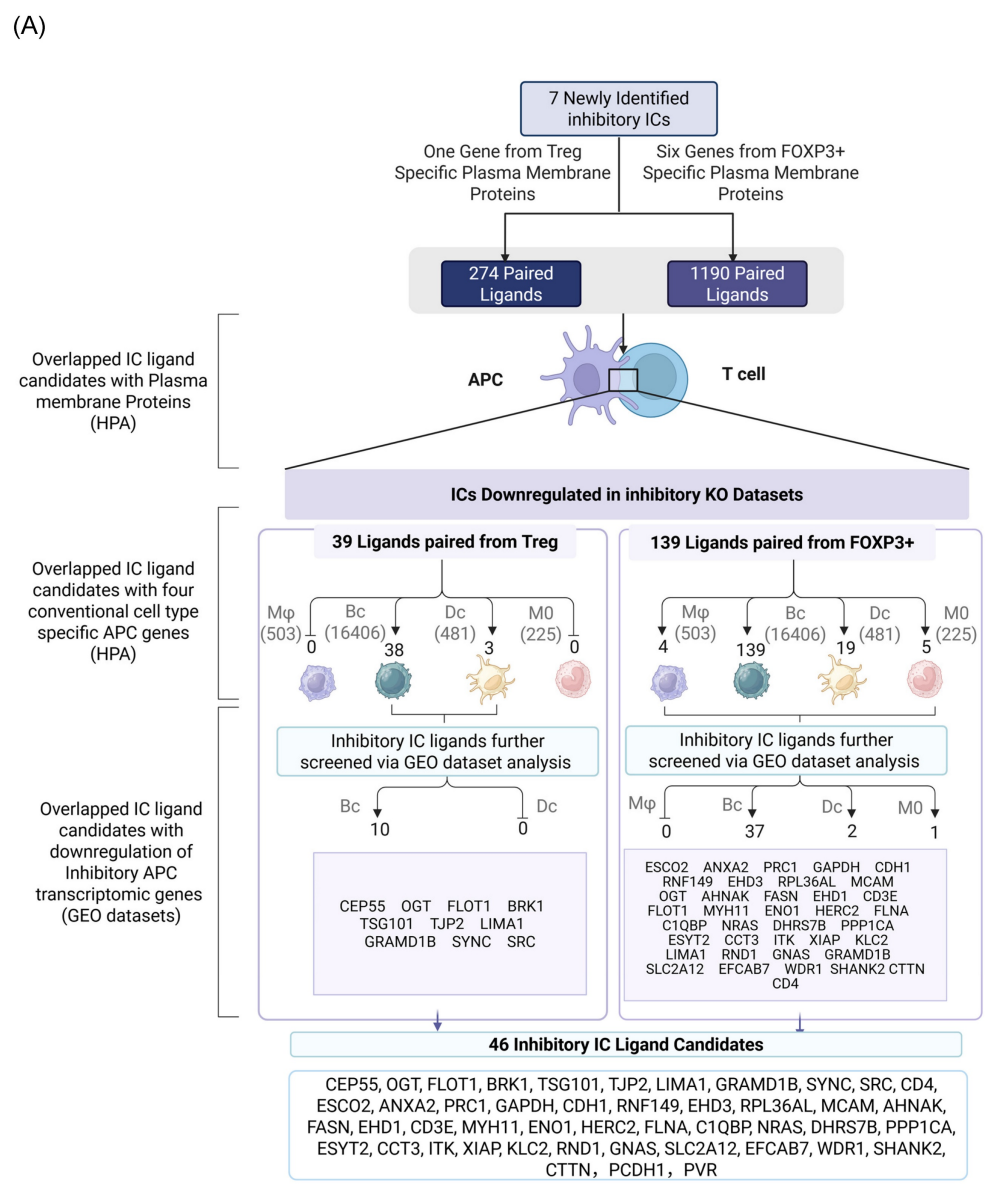

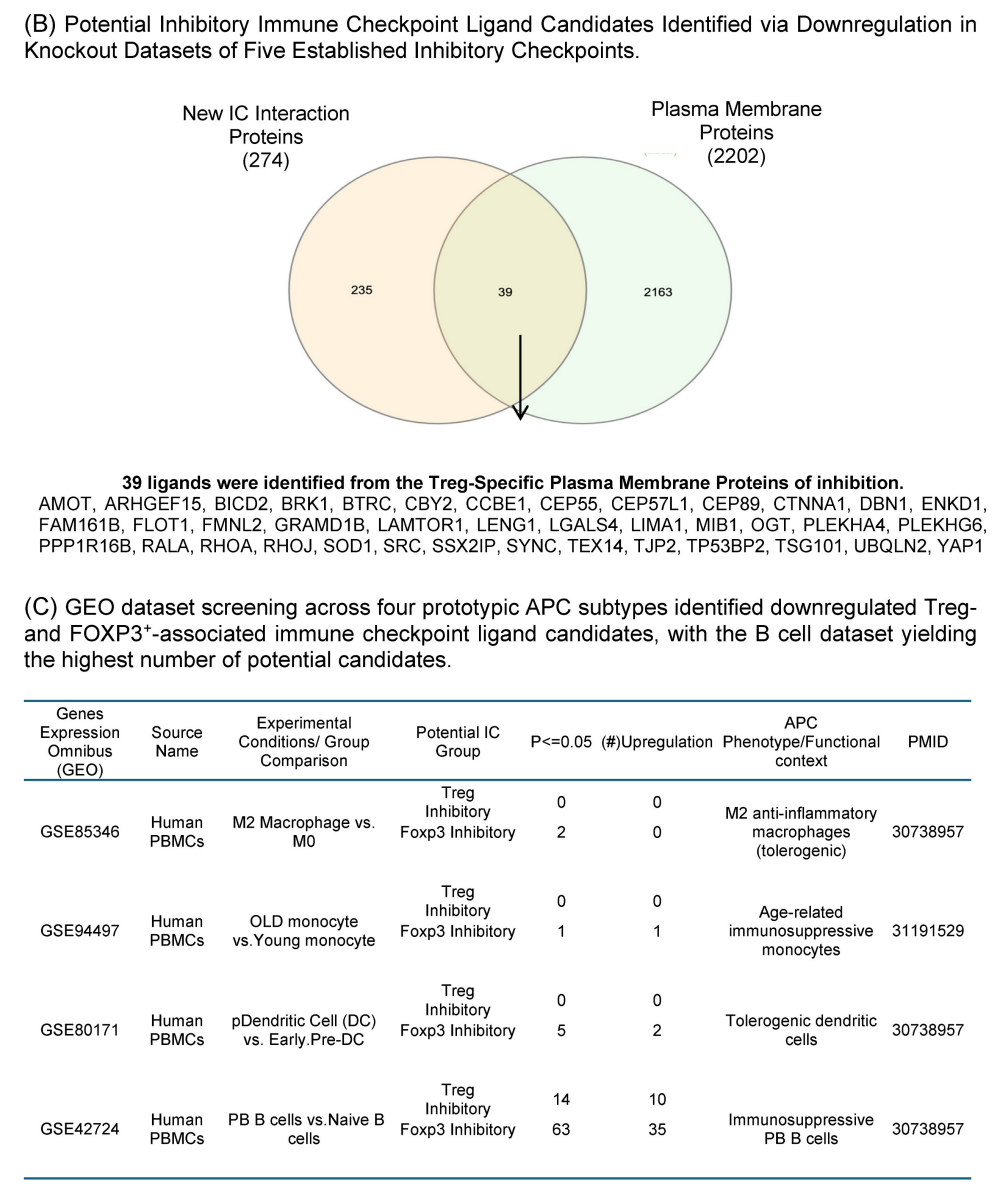

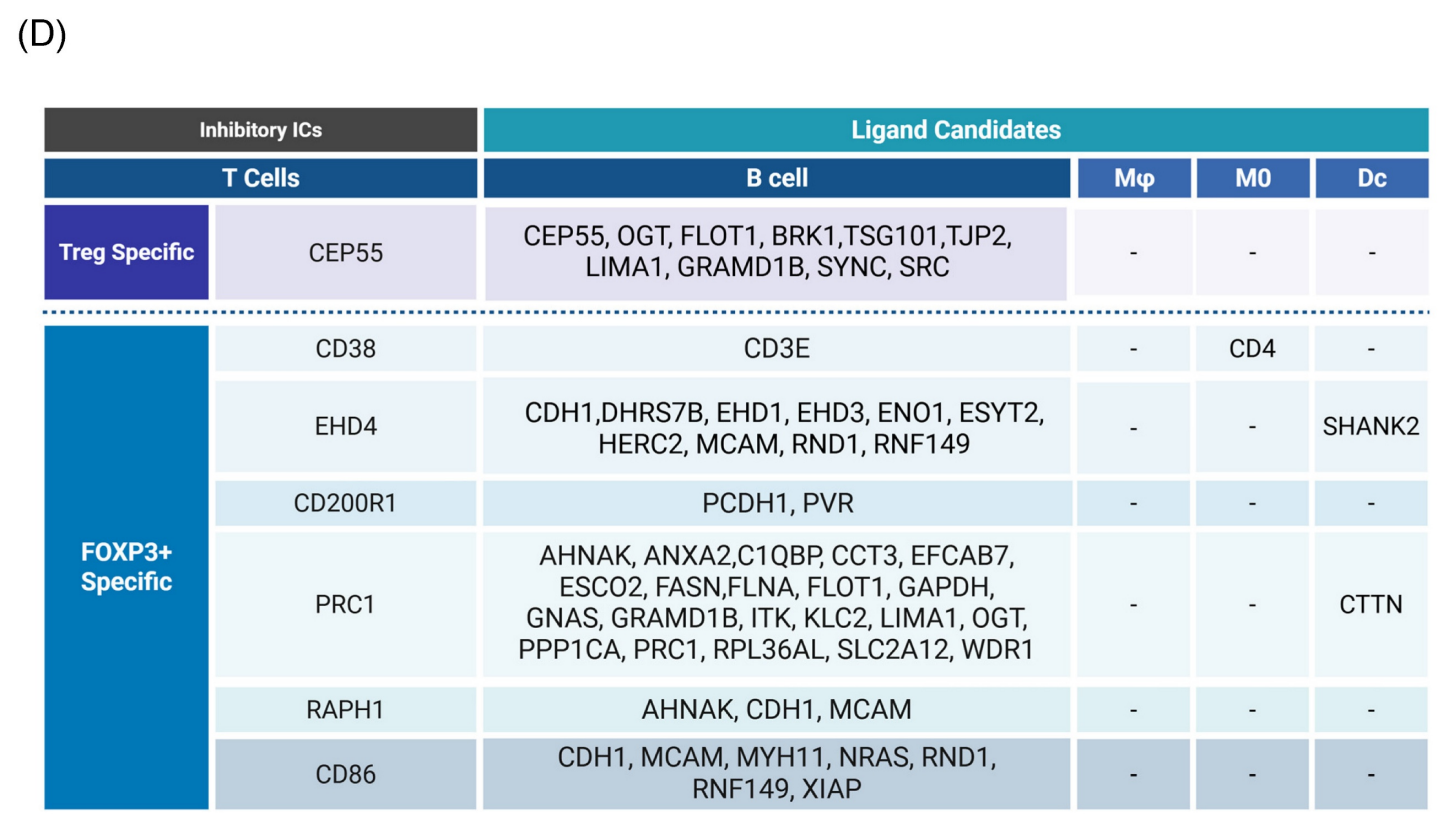
Identification of Novel Inhibitory IC ligands expressed on the plasma membrane of inhibitory antigen-presenting cells (APCs). (A) The flow chart illustrates the strategy used to identify novel immune checkpoints with inhibitory function and their corresponding paired ligands. The paired ligands were further overlapped with plasma membrane proteins and compared with marker genes from four traditional APC types: macrophages, B cells, dendritic cells, and monocytes. Specifically, we identified 39 and 139 ligands that were paired with Treg- and Foxp3⁺-specific immune checkpoints. This analysis revealed the distribution of these ligands across the four classical APC subsets. To assess their inhibitory potential, we further screened these ligands using GEO datasets enriched for genes upregulated during inhibitory APC function, narrowing the list to 46 unique inhibitory ligands. (B) We screened IC ligand candidate genes using 274 Treg-specific and 1,190 FOXP3⁺-specific paired ligands mapped to plasma membrane proteins. These were then overlapped with a reference list of 2,202 known human plasma membrane proteins. As a result, we identified 39 Treg-specific and 139 FOXP3⁺-specific potential inhibitory immune checkpoint ligands. (C) Summarizes the upregulation of candidate immune checkpoint ligands across multiple GEO datasets derived from human peripheral blood mononuclear cells. Each dataset compares tolerogenic or immunosuppressive APC subsets—including M2a macrophages, plasmacytoid dendritic cells (pDCs), PB B cells, and aged monocytes—with their respective control populations. Ligand candidates were grouped based on their potential association with Treg or FOXP3⁺ T cells and their predicted inhibitory role. PMIDs listed support the immunotolerogenic function of each APC type, justifying their use for identifying novel inhibitory IC ligands. (D) Summary of 7 newly identified ICs with their corresponding ligand candidates.

We hypothesized that 7 new ICs have their binding ligands expressed on the membrane of professional antigen presenting cells (APCs) to fulfill their immune suppressive functions via protein-protein interaction. To examine the hypothesis, we searched the experimental data of protein interactions in the NIH-NCBI-Gene database in order to identify protein binding ligands for new ICs (Fig. 4A). Then, new IC ligand candidates have to go through three rounds of screen including: *a)* human plasma membrane proteins; *b)* cell type specific proteins of four professional APC types such as macrophages (Mφ), B cells (Bc), dendritic cells (DCs), and monocytes (MOs); and *c)* expressions in inhibitory/immunotolerogenic subpopulations of macrophages, B cells, DC, and monocytes. As shown in Fig. 4A, 274 interaction protein genes were identified for IC ligand candidates for Treg derived new inhibitory IC CEP55, and 1,190 interaction protein genes were identified for 6 new ICs from Foxp3-upregulated new inhibitory ICs including CD38, EHD4, CD200R1, PRC1, RAPH1, and CD86.

As we outlined for the strategy with three steps in identifying new IC ligands in Fig. 2B, firstly, we screened for their localization on plasma membrane with the list of 2202 HPA plasma membrane proteins in Fig. 1A, 39 membrane protein ligands were identified from 274 CEP55 ligand candidates, and 139 ligand candidates for six new ICs CD38, EHD4, CD200R1, PRC1, RAPH1, and CD86 were identified from 1,190 ligand candidates after the first screen (Fig. 4B). Secondly, we screened IC ligand candidates for their expression potentials in four types of professional APCs with 503 HPA macrophage specific marker genes, 16,406 HPA B cell-specific marker genes, 481 HPA dendritic cell-specific marker genes and 225 HPA monocyte-specific marker genes. As shown in Fig. 4A, 41 IC ligand candidates were identified for CEP55, and 167 IC ligand candidates were identified for six new ICs CD38, EHD4, CD200R1, PRC1, RAPH1, and CD86 after the second screen. Thirdly, we screened 208 IC ligands derived from the previous two screens for their preferred expressions in inhibitory/immunosuppressive subpopulations (four GeoDatasets in Fig. 4C) of four professional APCs including human anti-inflammatory M2 macropahges 57, human immune suppressive B1 B cells, human immunotolerogenic DCs 57 and anti-inflammatory monocytes 58. Finally, 10 new IC ligands (10 including potential CEP55 homodimer) were identified for CEP55, and 40 new IC ligands were identified for six new ICs CD38, EHD4, CD200R1, PRC1, RAPH1, and CD86. After removing the 3 duplicates, 46 new inhibitory IC ligands were identified for 7 new ICs (Fig. 4D), which included 10 ligands from B cells for CEP55, two ligands from B cells for CD38, 11 ligands from B cells and DCs for EHD4, two ligands from B cells for CD200R1, 22 ligands from B cells and DCs for PRC1, three ligands from B cells for RAPH1, and 7 ligands from B cells for CD86. These results demonstrate that each ICs expressed on T cells/Treg have multiple ligands expressed on APCs that mediate multiple intercellular interactions in different pathophysiological contexts; and B cells as a prototypic APC contribute significantly to immunosuppression and signaling pathways carried out by new inhibitory ICs and IC ligands. Our finding on multiple IC ligands for each of the new ICs were well correlated with the experimental data for multiple binding partners of 5 best characterized high hierarchy ICs such as CTLA4, KLRG1, LAG3, PD-1 and TIGIT in the NIH-NCBI-Gene database. The future work is needed to certify these IC interaction proteins as known IC ligands via going through vigorous 3 rounds of screening for plasma membrane localization, specific expression in professional APCs and preferred expression in inhibitory/immune tolerogenic subpopulations of professional APCs using our new strategy as we demonstrated here.

### 6. Integrated regulation of seven newly identified inhibitory ICs and their 46 ligands across tumor, T cell activation, and redox contexts

To assess the biological relevance of the seven newly identified inhibitory immune checkpoints, we examined their expression dynamics in activated T cells, tumor-infiltrating Tregs, and cells with altered redox regulation. Analysis of multiple GEO transcriptomic datasets revealed that both classical and newly identified ICs were modulated during T-cell activation, indicating that inhibitory checkpoint expression is dynamically regulated in response to immune stimulation.

Across 11 datasets of tumor-derived Tregs from various cancers, all seven new ICs were consistently upregulated, mirroring expression trends observed in 25 well-characterized ICs. The strong positive correlation between known and novel ICs in tumor Tregs suggests that these molecules contribute to the immunosuppressive phenotype of tumor-associated Tregs and may facilitate immune evasion within the tumor microenvironment. We next explored whether redox-related transcriptional pathways influence IC expression. In datasets with deficiencies of the antioxidant transcription factor NRF2 and other ROS-regulating factors, all seven new ICs and a large subset of known ICs were significantly modulated, with most showing increased expression. Similarly, analysis of 46 newly identified IC ligands revealed that their expression was broadly regulated by ROS-associated transcription factors. Notably, 30 of the 46 ligands (65%) were significantly altered in PD-L1 knock-down models, further underscoring their functional connection to established immunosuppressive networks (Figure S7-12). Collectively, these integrated analyses demonstrate that the newly identified ICs and their ligands are not only upregulated in tumor Tregs but also dynamically modulated by immune activation and redox signaling.

## Discussion

Immune checkpoint inhibitors (ICIs) have revolutionized cancer treatment, significantly improving outcomes across a range of malignancies. However, their clinical success is often limited by immune-related adverse events (irAEs) 59, highlighting the need to better understand the full network of immune regulatory mechanisms. One major gap is incomplete understanding of the diverse membrane proteins expressed by Tregs, especially those regulated by the Treg-specific transcription factor Foxp3, and how these proteins interact with ligands expressed by inhibitory APCs. To address those questions, we developed a comprehensive, multi-step screening strategy that integrates the experimental data from transcriptomic, proteomic, and systems biology datasets to identify novel inhibitory ICs and their ligands. In the first round of screening, we identified 151 candidate ICs, comprising 45 Treg-specific membrane proteins and 106 Foxp3-upregulated membrane proteins. These candidates were benchmarked against the 10 best-characterized inhibitory ICs, including CTLA4, PDCD1 (PD-1), LAG3, TIGIT, KLRG1, and others based on frequency in the literature and functional evidence. In the second round, we selected IC candidates that were significantly downregulated in at least one of the 10 IC-deficient or IC-blockade models, narrowing the list to 85 prioritized ICs. In the third and most stringent screening step, we further refined this list by identifying candidates downregulated in three or more of five well-characterized IC-deficient transcriptomic datasets, which led to the discovery of seven high-confidence novel ICs: CEP55, CD38, EHD4, CD200R1, PRC1, RAPH1, and CD86. Using scRNA-seq, we confirmed that these seven ICs are broadly expressed in T cell subsets, including CD4⁺ T cells, CD8⁺ T cells, mitotic and tissue-resident T cells, as well as Tregs. Notably, their expression patterns closely mirrored those of canonical inhibitory ICs, suggesting potential immunosuppressive roles.

To identify ligands for these novel ICs, we applied a three-step filter: (1) localization to the plasma membrane, (2) expression in professional APCs (monocytes, macrophages, dendritic cells, and B cells), and (3) preferential expression in anti-inflammatory or tolerogenic APC subpopulations. This strategy yielded 46 novel inhibitory IC ligands. Functionally, both the seven novel ICs and the canonical ICs were significantly upregulated in tumor-infiltrating Tregs, suggesting they contribute to Treg-mediated immunosuppression within the tumor microenvironment. In contrast, their expression in activated effector T cells varied, indicating cell-type and context-dependent regulation.

**Figure 5:**
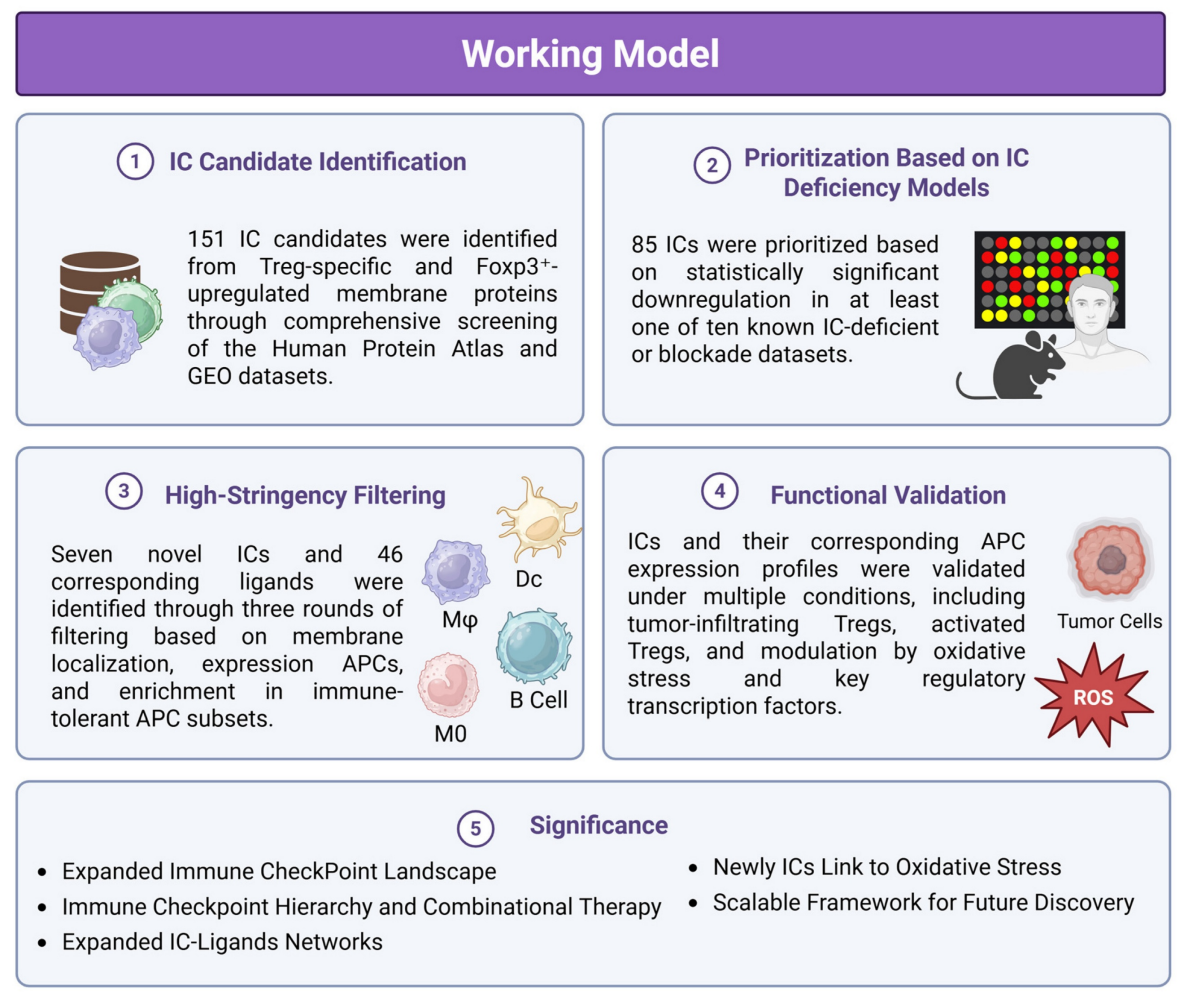
This schematic illustrates a multi-step strategy for discovering novel inhibitory ICs and their ligands using integrated transcriptomic, proteomic, and systems biology approaches. (1) IC Candidate Identification: A total of 151 IC candidates were identified from Treg-specific and Foxp3⁺-upregulated membrane proteins through comprehensive screening of the Human Protein Atlas and GEO datasets. (2) Prioritization Based on IC Deficiency Models: 85 ICs were prioritized based on statistically significant downregulation in at least one of ten IC-deficient or blockade transcriptomic datasets. (3) High-Stringency Filtering: Seven novel ICs and 46 ligands were further refined through three rounds of filtering based on plasma membrane localization, expression in professional antigen-presenting cells (APCs), and enrichment in immune-tolerant APC subsets. (4) Functional Validation: Expression of ICs and their ligands was validated across multiple conditions, including tumor-infiltrating Tregs, activated Tregs, and under oxidative stress, showing modulation by key regulatory transcription factors. (5) Significance: This workflow expands the known IC landscape and ligand networks, reveals a potential regulatory hierarchy among ICs, and identifies redox-sensitive IC pathways. The strategy provides a scalable framework for future immunotherapy development and disease classification.

These findings address several important knowledge gaps (Fig 5): 1) Expanded Immune Checkpoint Landscape: Our study broadens the known repertoire of Treg-expressed immune checkpoints. While 46 ICs are currently described in the literature, our work identifies 151 additional candidates, with 85 prioritized and 7 validated through high-stringency screenings. These may support the classification of additional Treg subpopulations and clarify their roles in diverse pathological contexts. 2) Immune Checkpoint Hierarchy and Combinational Therapy: We demonstrate for the first time that certain ICs, such as CTLA4, PD-1, LAG3, TIGIT, and KLRG1, exhibit regulatory control over other ICs, placing them in a high-hierarchy group. This regulatory relationship is critical for optimizing combinational ICI therapy, which may be more effective than current monotherapies that benefit only ~20% of patients 48. 3) Expanded IC-Ligand Networks: While some ICs like TIGIT are known to interact with multiple ligands, our data identify 46 new ligands that bind to the 7 novel ICs. This significantly expands the IC-ligand network and offers new targets for immunotherapy, especially in tumors resistant to conventional ICIs 60, 61. 4) Link to Oxidative Stress and Metabolism: Our findings demonstrate that immune checkpoint expression is closely tied to redox and metabolic signaling. The modulation of ICs and their ligands by NRF2 and related transcription factors highlights a mechanistic link between oxidative stress and immune regulation, which may be relevant in cancer, chronic inflammation, and metabolic disease. 5) Scalable Framework for Future Discovery: Our multi-dimensional approach combining curated gene expression datasets, plasma membrane protein localization, gene knockout data, and protein-protein interaction networks can serve as a generalizable framework for identifying other functional regulatory systems in immunity and disease.

In summary, our study identifies new immune checkpoints and ligands with immunosuppressive potential, reveals new mechanisms of immune regulation, and proposes therapeutic strategies that could enhance the efficacy of cancer immunotherapy and treat a wide range of immune-mediated diseases.

## Supplementary Material

Supplementary figures.

Supplementary table 1.
