# Supplementary material for: Discovery of Seven ROS-Sensitive Immune Checkpoints and 46 Ligands Mediating Immune Suppression Through T cell-APC Networks
Source: J Cancer. 2026 Jan 14;17(2):439–56. doi: 10.7150/jca.128083 (PMC12825432; doi:10.7150/jca.128083)
Supplement: Supplementary file 1 — Supplementary figures. [file jcav17p0439s1.pptx]

## Slide 1
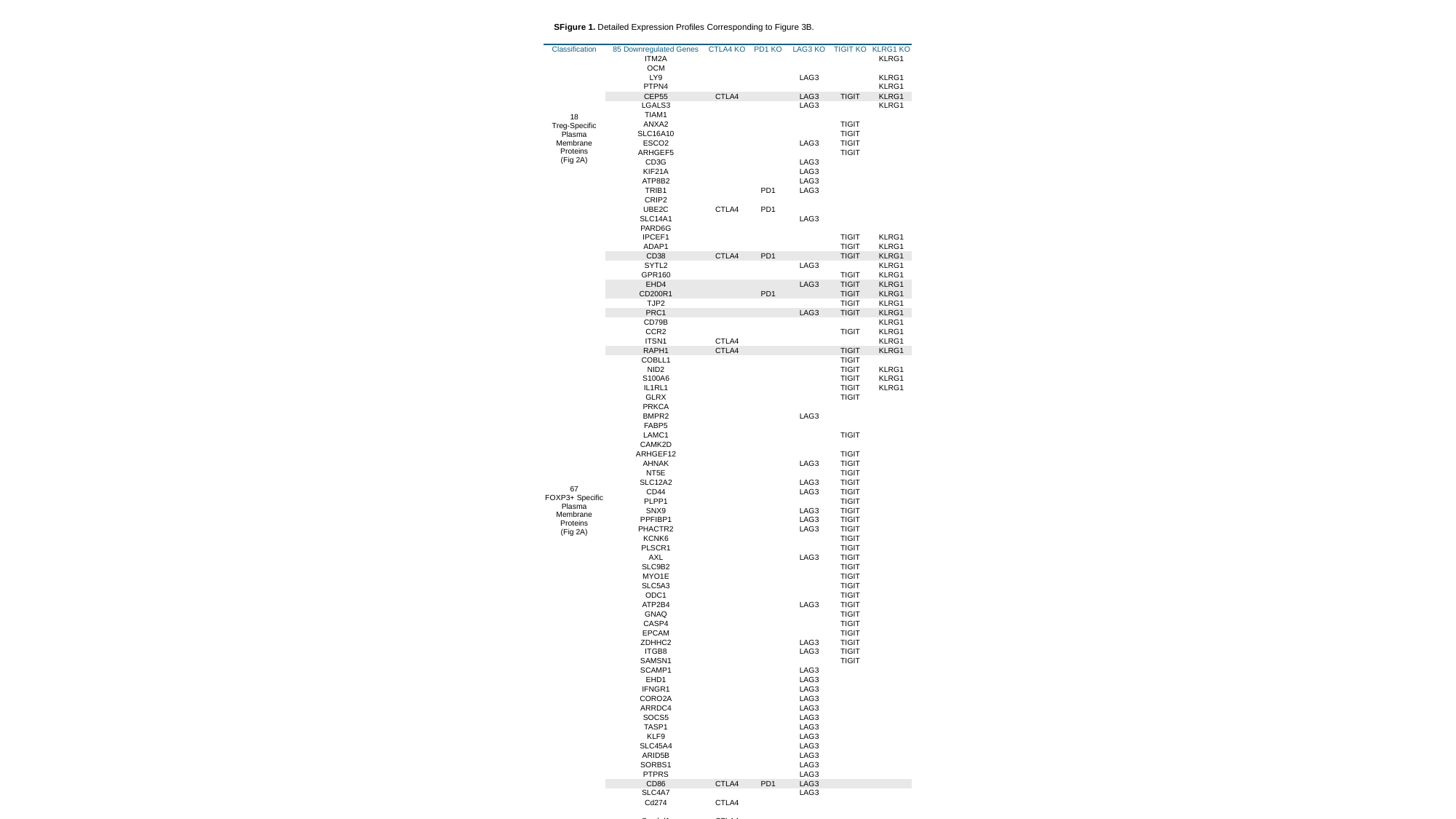

SFigure 1. Detailed Expression Profiles Corresponding to Figure 3B.
| Classification | 85 Downregulated Genes | CTLA4 KO | PD1 KO | LAG3 KO | TIGIT KO | KLRG1 KO |
| --- | --- | --- | --- | --- | --- | --- |
| 18 Treg-Specific Plasma Membrane Proteins (Fig 2A) | ITM2A | | | | | KLRG1 |
| | OCM | | | | | |
| | LY9 | | | LAG3 | | KLRG1 |
| | PTPN4 | | | | | KLRG1 |
| | CEP55 | CTLA4 | | LAG3 | TIGIT | KLRG1 |
| | LGALS3 | | | LAG3 | | KLRG1 |
| | TIAM1 | | | | | |
| | ANXA2 | | | | TIGIT | |
| | SLC16A10 | | | | TIGIT | |
| | ESCO2 | | | LAG3 | TIGIT | |
| | ARHGEF5 | | | | TIGIT | |
| | CD3G | | | LAG3 | | |
| | KIF21A | | | LAG3 | | |
| | ATP8B2 | | | LAG3 | | |
| | TRIB1 | | PD1 | LAG3 | | |
| | CRIP2 | | | | | |
| | UBE2C | CTLA4 | PD1 | | | |
| | SLC14A1 | | | LAG3 | | |
| 67 FOXP3+ Specific Plasma Membrane Proteins (Fig 2A) | PARD6G | | | | | |
| | IPCEF1 | | | | TIGIT | KLRG1 |
| | ADAP1 | | | | TIGIT | KLRG1 |
| | CD38 | CTLA4 | PD1 | | TIGIT | KLRG1 |
| | SYTL2 | | | LAG3 | | KLRG1 |
| | GPR160 | | | | TIGIT | KLRG1 |
| | EHD4 | | | LAG3 | TIGIT | KLRG1 |
| | CD200R1 | | PD1 | | TIGIT | KLRG1 |
| | TJP2 | | | | TIGIT | KLRG1 |
| | PRC1 | | | LAG3 | TIGIT | KLRG1 |
| | CD79B | | | | | KLRG1 |
| | CCR2 | | | | TIGIT | KLRG1 |
| | ITSN1 | CTLA4 | | | | KLRG1 |
| | RAPH1 | CTLA4 | | | TIGIT | KLRG1 |
| | COBLL1 | | | | TIGIT | |
| | NID2 | | | | TIGIT | KLRG1 |
| | S100A6 | | | | TIGIT | KLRG1 |
| | IL1RL1 | | | | TIGIT | KLRG1 |
| | GLRX | | | | TIGIT | |
| | PRKCA | | | | | |
| | BMPR2 | | | LAG3 | | |
| | FABP5 | | | | | |
| | LAMC1 | | | | TIGIT | |
| | CAMK2D | | | | | |
| | ARHGEF12 | | | | TIGIT | |
| | AHNAK | | | LAG3 | TIGIT | |
| | NT5E | | | | TIGIT | |
| | SLC12A2 | | | LAG3 | TIGIT | |
| | CD44 | | | LAG3 | TIGIT | |
| | PLPP1 | | | | TIGIT | |
| | SNX9 | | | LAG3 | TIGIT | |
| | PPFIBP1 | | | LAG3 | TIGIT | |
| | PHACTR2 | | | LAG3 | TIGIT | |
| | KCNK6 | | | | TIGIT | |
| | PLSCR1 | | | | TIGIT | |
| | AXL | | | LAG3 | TIGIT | |
| | SLC9B2 | | | | TIGIT | |
| | MYO1E | | | | TIGIT | |
| | SLC5A3 | | | | TIGIT | |
| | ODC1 | | | | TIGIT | |
| | ATP2B4 | | | LAG3 | TIGIT | |
| | GNAQ | | | | TIGIT | |
| | CASP4 | | | | TIGIT | |
| | EPCAM | | | | TIGIT | |
| | ZDHHC2 | | | LAG3 | TIGIT | |
| | ITGB8 | | | LAG3 | TIGIT | |
| | SAMSN1 | | | | TIGIT | |
| | SCAMP1 | | | LAG3 | | |
| | EHD1 | | | LAG3 | | |
| | IFNGR1 | | | LAG3 | | |
| | CORO2A | | | LAG3 | | |
| | ARRDC4 | | | LAG3 | | |
| | SOCS5 | | | LAG3 | | |
| | TASP1 | | | LAG3 | | |
| | KLF9 | | | LAG3 | | |
| | SLC45A4 | | | LAG3 | | |
| | ARID5B | | | LAG3 | | |
| | SORBS1 | | | LAG3 | | |
| | PTPRS | | | LAG3 | | |
| | CD86 | CTLA4 | PD1 | LAG3 | | |
| | SLC4A7 | | | LAG3 | | |
| | Cd274 | CTLA4 | | | | |
| | Samhd1 | CTLA4 | | | | |
| | Snap23 | CTLA4 | | | | |

## Slide 2
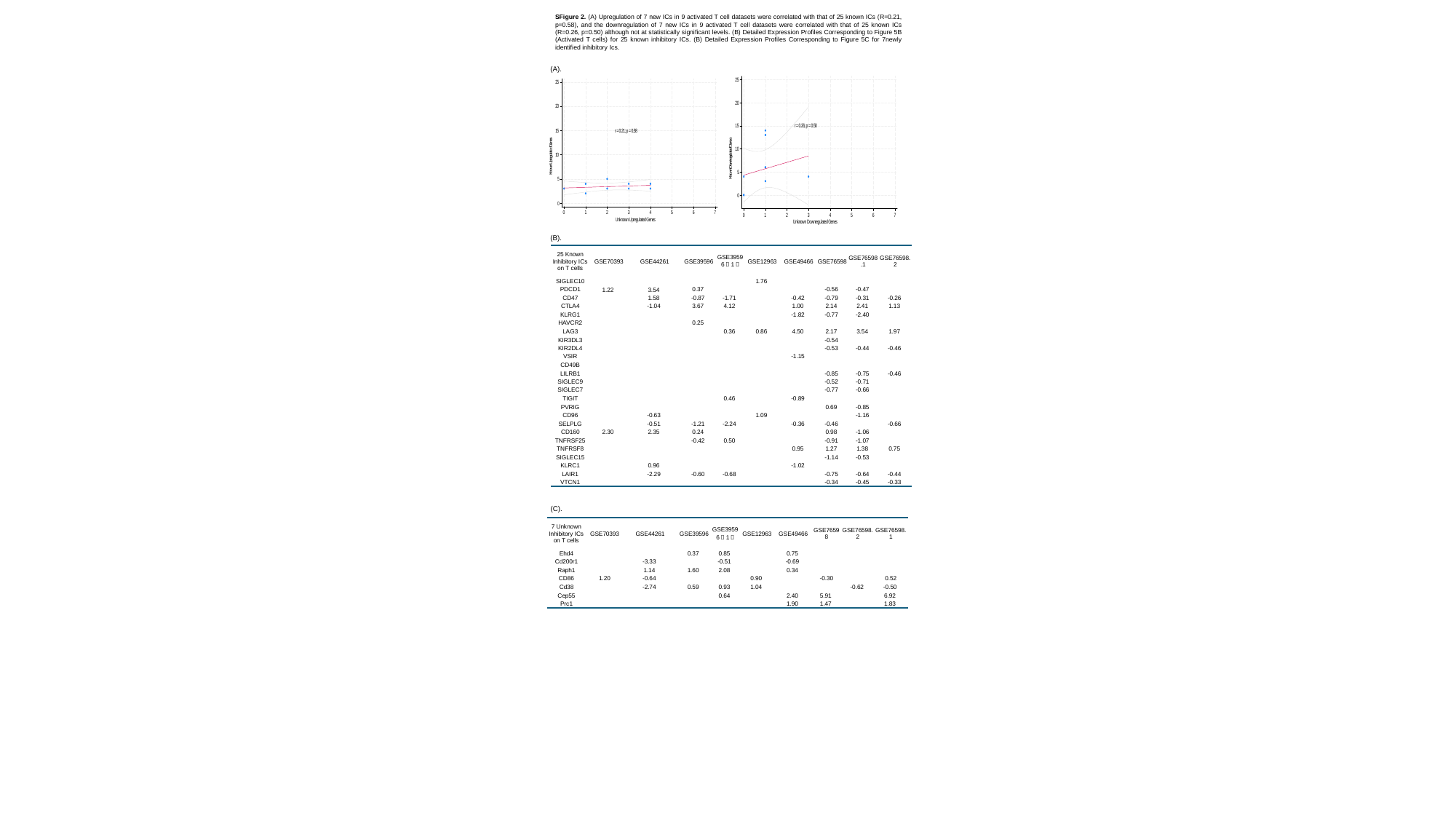

SFigure 2. (A) Upregulation of 7 new ICs in 9 activated T cell datasets were correlated with that of 25 known ICs (R=0.21, p=0.58), and the downregulation of 7 new ICs in 9 activated T cell datasets were correlated with that of 25 known ICs (R=0.26, p=0.50) although not at statistically significant levels. (B) Detailed Expression Profiles Corresponding to Figure 5B (Activated T cells) for 25 known inhibitory ICs. (B) Detailed Expression Profiles Corresponding to Figure 5C for 7newly identified inhibitory Ics.
(A).
(B).
| 25 Known Inhibitory ICs on T cells | GSE70393 | GSE44261 | GSE39596 | GSE39596（1） | GSE12963 | GSE49466 | GSE76598 | GSE76598.1 | GSE76598.2 |
| --- | --- | --- | --- | --- | --- | --- | --- | --- | --- |
| SIGLEC10 | | | | | 1.76 | | | | |
| PDCD1 | 1.22 | 3.54 | 0.37 | | | | -0.56 | -0.47 | |
| CD47 | | 1.58 | -0.87 | -1.71 | | -0.42 | -0.79 | -0.31 | -0.26 |
| CTLA4 | | -1.04 | 3.67 | 4.12 | | 1.00 | 2.14 | 2.41 | 1.13 |
| KLRG1 | | | | | | -1.82 | -0.77 | -2.40 | |
| HAVCR2 | | | 0.25 | | | | | | |
| LAG3 | | | | 0.36 | 0.86 | 4.50 | 2.17 | 3.54 | 1.97 |
| KIR3DL3 | | | | | | | -0.54 | | |
| KIR2DL4 | | | | | | | -0.53 | -0.44 | -0.46 |
| VSIR | | | | | | -1.15 | | | |
| CD49B | | | | | | | | | |
| LILRB1 | | | | | | | -0.85 | -0.75 | -0.46 |
| SIGLEC9 | | | | | | | -0.52 | -0.71 | |
| SIGLEC7 | | | | | | | -0.77 | -0.66 | |
| TIGIT | | | | 0.46 | | -0.89 | | | |
| PVRIG | | | | | | | 0.69 | -0.85 | |
| CD96 | | -0.63 | | | 1.09 | | | -1.16 | |
| SELPLG | | -0.51 | -1.21 | -2.24 | | -0.36 | -0.46 | | -0.66 |
| CD160 | 2.30 | 2.35 | 0.24 | | | | 0.98 | -1.06 | |
| TNFRSF25 | | | -0.42 | 0.50 | | | -0.91 | -1.07 | |
| TNFRSF8 | | | | | | 0.95 | 1.27 | 1.38 | 0.75 |
| SIGLEC15 | | | | | | | -1.14 | -0.53 | |
| KLRC1 | | 0.96 | | | | -1.02 | | | |
| LAIR1 | | -2.29 | -0.60 | -0.68 | | | -0.75 | -0.64 | -0.44 |
| VTCN1 | | | | | | | -0.34 | -0.45 | -0.33 |
(C).
| 7 Unknown Inhibitory ICs on T cells | GSE70393 | GSE44261 | GSE39596 | GSE39596（1） | GSE12963 | GSE49466 | GSE76598 | GSE76598.2 | GSE76598.1 |
| --- | --- | --- | --- | --- | --- | --- | --- | --- | --- |
| Ehd4 | | | 0.37 | 0.85 | | 0.75 | | | |
| Cd200r1 | | -3.33 | | -0.51 | | -0.69 | | | |
| Raph1 | | 1.14 | 1.60 | 2.08 | | 0.34 | | | |
| CD86 | 1.20 | -0.64 | | | 0.90 | | -0.30 | | 0.52 |
| Cd38 | | -2.74 | 0.59 | 0.93 | 1.04 | | | -0.62 | -0.50 |
| Cep55 | | | | 0.64 | | 2.40 | 5.91 | | 6.92 |
| Prc1 | | | | | | 1.90 | 1.47 | | 1.83 |

## Slide 3
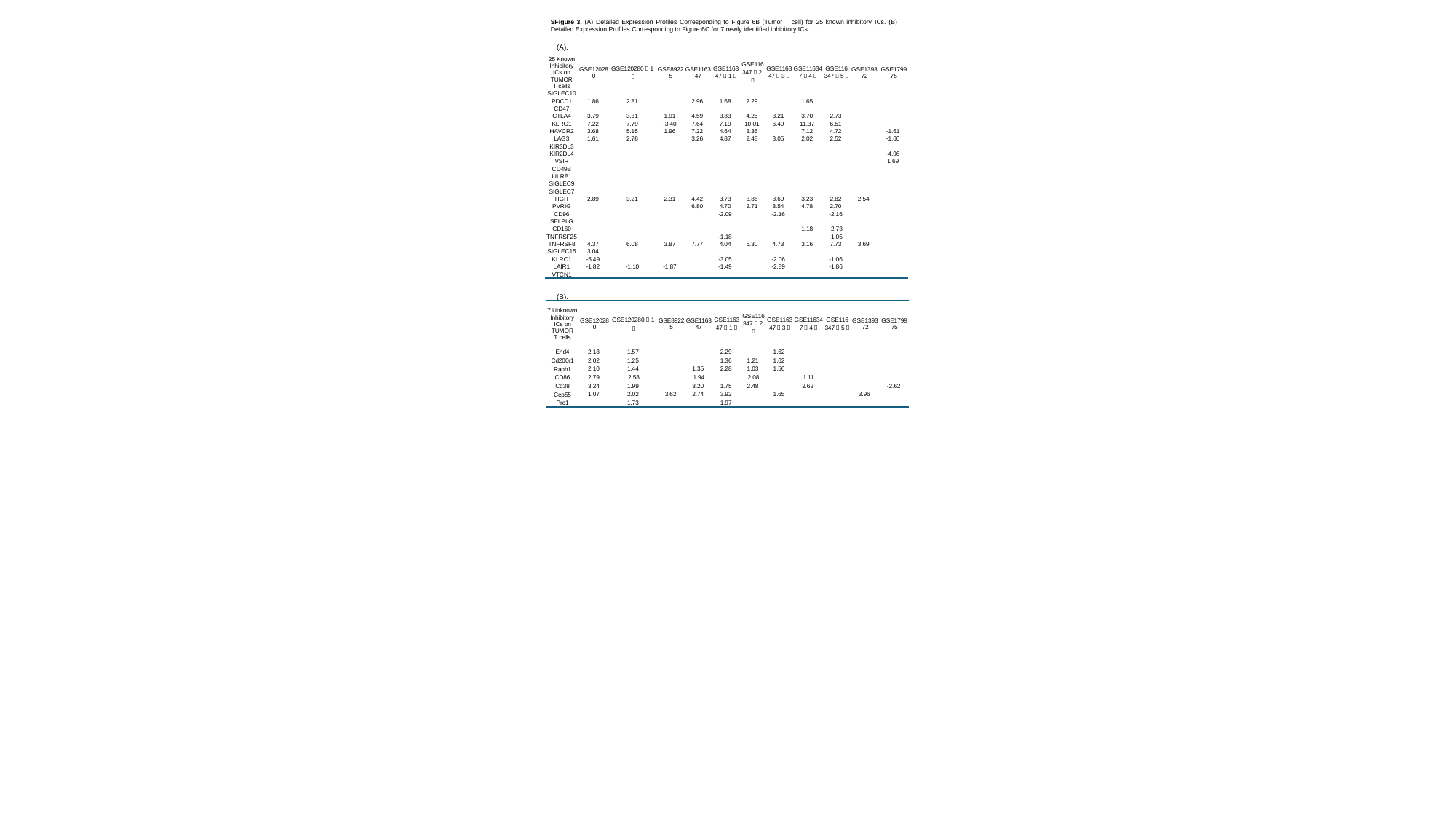

SFigure 3. (A) Detailed Expression Profiles Corresponding to Figure 6B (Tumor T cell) for 25 known inhibitory ICs. (B) Detailed Expression Profiles Corresponding to Figure 6C for 7 newly identified inhibitory ICs.
(A).
| 25 Known Inhibitory ICs on TUMOR T cells | GSE120280 | GSE120280（1） | GSE89225 | GSE116347 | GSE116347（1） | GSE116347（2） | GSE116347（3） | GSE116347（4） | GSE116347（5） | GSE139372 | GSE179975 |
| --- | --- | --- | --- | --- | --- | --- | --- | --- | --- | --- | --- |
| SIGLEC10 | | | | | | | | | | | |
| PDCD1 | 1.86 | 2.81 | | 2.96 | 1.68 | 2.29 | | 1.65 | | | |
| CD47 | | | | | | | | | | | |
| CTLA4 | 3.79 | 3.31 | 1.91 | 4.59 | 3.83 | 4.25 | 3.21 | 3.70 | 2.73 | | |
| KLRG1 | 7.22 | 7.79 | -3.40 | 7.64 | 7.19 | 10.01 | 6.49 | 11.37 | 6.51 | | |
| HAVCR2 | 3.68 | 5.15 | 1.96 | 7.22 | 4.64 | 3.35 | | 7.12 | 4.72 | | -1.61 |
| LAG3 | 1.61 | 2.78 | | 3.26 | 4.87 | 2.48 | 3.05 | 2.02 | 2.52 | | -1.60 |
| KIR3DL3 | | | | | | | | | | | |
| KIR2DL4 | | | | | | | | | | | -4.96 |
| VSIR | | | | | | | | | | | 1.69 |
| CD49B | | | | | | | | | | | |
| LILRB1 | | | | | | | | | | | |
| SIGLEC9 | | | | | | | | | | | |
| SIGLEC7 | | | | | | | | | | | |
| TIGIT | 2.89 | 3.21 | 2.31 | 4.42 | 3.73 | 3.86 | 3.69 | 3.23 | 2.82 | 2.54 | |
| PVRIG | | | | 6.80 | 4.70 | 2.71 | 3.54 | 4.78 | 2.70 | | |
| CD96 | | | | | -2.09 | | -2.16 | | -2.16 | | |
| SELPLG | | | | | | | | | | | |
| CD160 | | | | | | | | 1.18 | -2.73 | | |
| TNFRSF25 | | | | | -1.18 | | | | -1.05 | | |
| TNFRSF8 | 4.37 | 6.08 | 3.87 | 7.77 | 4.04 | 5.30 | 4.73 | 3.16 | 7.73 | 3.69 | |
| SIGLEC15 | 3.04 | | | | | | | | | | |
| KLRC1 | -5.49 | | | | -3.05 | | -2.06 | | -1.06 | | |
| LAIR1 | -1.82 | -1.10 | -1.87 | | -1.49 | | -2.89 | | -1.86 | | |
| VTCN1 | | | | | | | | | | | |
(B).
| 7 Unknown Inhibitory ICs on TUMOR T cells | GSE120280 | GSE120280（1） | GSE89225 | GSE116347 | GSE116347（1） | GSE116347（2） | GSE116347（3） | GSE116347（4） | GSE116347（5） | GSE139372 | GSE179975 |
| --- | --- | --- | --- | --- | --- | --- | --- | --- | --- | --- | --- |
| Ehd4 | 2.18 | 1.57 | | | 2.29 | | 1.62 | | | | |
| Cd200r1 | 2.02 | 1.25 | | | 1.36 | 1.21 | 1.62 | | | | |
| Raph1 | 2.10 | 1.44 | | 1.35 | 2.28 | 1.03 | 1.56 | | | | |
| CD86 | 2.79 | 2.58 | | 1.94 | | 2.08 | | 1.11 | | | |
| Cd38 | 3.24 | 1.99 | | 3.20 | 1.75 | 2.48 | | 2.62 | | | -2.62 |
| Cep55 | 1.07 | 2.02 | 3.62 | 2.74 | 3.92 | | 1.65 | | | 3.96 | |
| Prc1 | | 1.73 | | | 1.97 | | | | | | |

## Slide 4
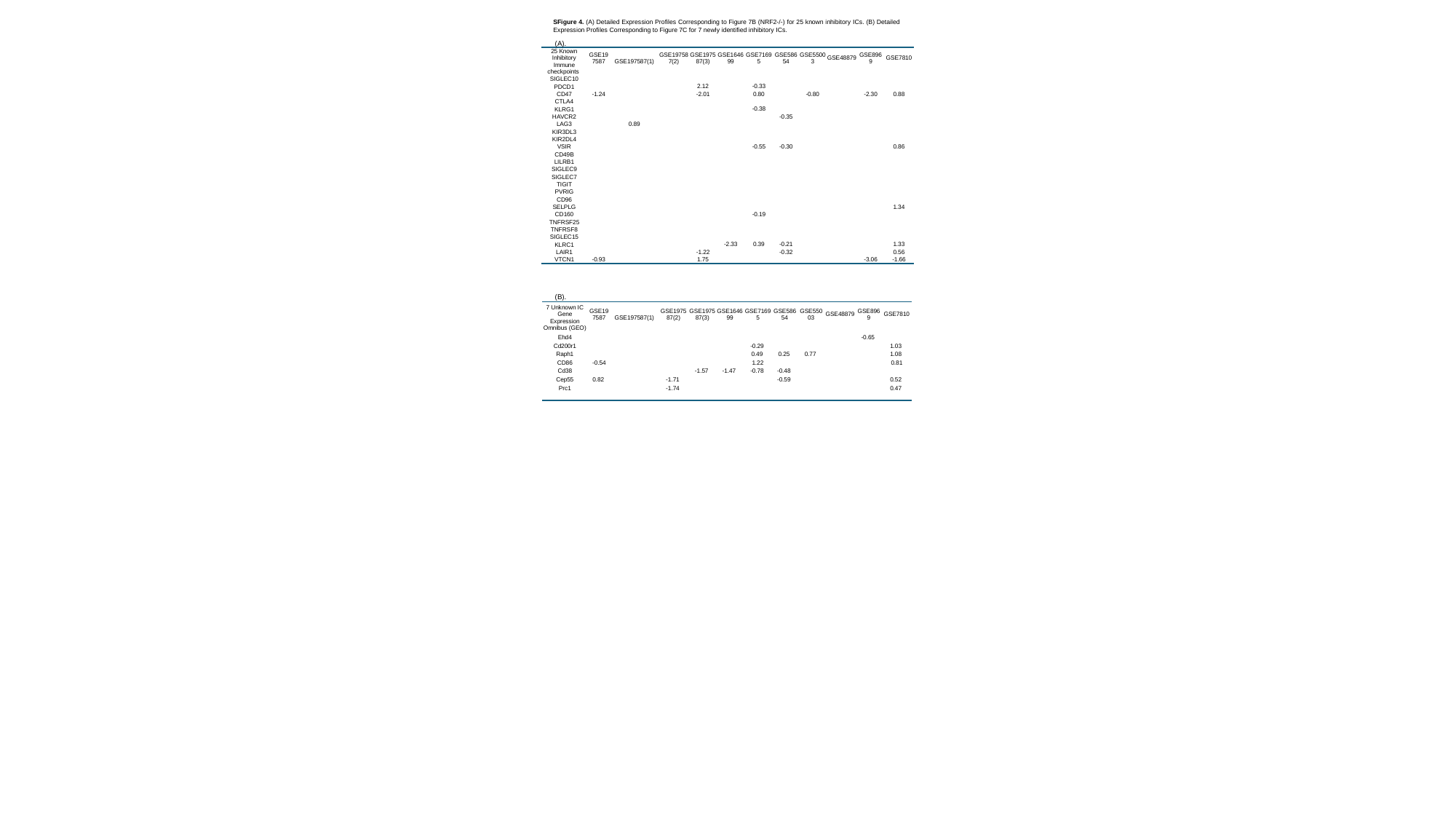

SFigure 4. (A) Detailed Expression Profiles Corresponding to Figure 7B (NRF2-/-) for 25 known inhibitory ICs. (B) Detailed Expression Profiles Corresponding to Figure 7C for 7 newly identified inhibitory ICs.
(A).
| 25 Known Inhibitory Immune checkpoints | GSE197587 | GSE197587(1) | GSE197587(2) | GSE197587(3) | GSE164699 | GSE71695 | GSE58654 | GSE55003 | GSE48879 | GSE8969 | GSE7810 |
| --- | --- | --- | --- | --- | --- | --- | --- | --- | --- | --- | --- |
| SIGLEC10 | | | | | | | | | | | |
| PDCD1 | | | | 2.12 | | -0.33 | | | | | |
| CD47 | -1.24 | | | -2.01 | | 0.80 | | -0.80 | | -2.30 | 0.88 |
| CTLA4 | | | | | | | | | | | |
| KLRG1 | | | | | | -0.38 | | | | | |
| HAVCR2 | | | | | | | -0.35 | | | | |
| LAG3 | | 0.89 | | | | | | | | | |
| KIR3DL3 | | | | | | | | | | | |
| KIR2DL4 | | | | | | | | | | | |
| VSIR | | | | | | -0.55 | -0.30 | | | | 0.86 |
| CD49B | | | | | | | | | | | |
| LILRB1 | | | | | | | | | | | |
| SIGLEC9 | | | | | | | | | | | |
| SIGLEC7 | | | | | | | | | | | |
| TIGIT | | | | | | | | | | | |
| PVRIG | | | | | | | | | | | |
| CD96 | | | | | | | | | | | |
| SELPLG | | | | | | | | | | | 1.34 |
| CD160 | | | | | | -0.19 | | | | | |
| TNFRSF25 | | | | | | | | | | | |
| TNFRSF8 | | | | | | | | | | | |
| SIGLEC15 | | | | | | | | | | | |
| KLRC1 | | | | | -2.33 | 0.39 | -0.21 | | | | 1.33 |
| LAIR1 | | | | -1.22 | | | -0.32 | | | | 0.56 |
| VTCN1 | -0.93 | | | 1.75 | | | | | | -3.06 | -1.66 |
(B).
| 7 Unknown IC Gene Expression Omnibus (GEO) | GSE197587 | GSE197587(1) | GSE197587(2) | GSE197587(3) | GSE164699 | GSE71695 | GSE58654 | GSE55003 | GSE48879 | GSE8969 | GSE7810 |
| --- | --- | --- | --- | --- | --- | --- | --- | --- | --- | --- | --- |
| Ehd4 | | | | | | | | | | -0.65 | |
| Cd200r1 | | | | | | -0.29 | | | | | 1.03 |
| Raph1 | | | | | | 0.49 | 0.25 | 0.77 | | | 1.08 |
| CD86 | -0.54 | | | | | 1.22 | | | | | 0.81 |
| Cd38 | | | | -1.57 | -1.47 | -0.78 | -0.48 | | | | |
| Cep55 | 0.82 | | -1.71 | | | | -0.59 | | | | 0.52 |
| Prc1 | | | -1.74 | | | | | | | | 0.47 |
| | | | | | | | | | | | |

## Slide 5
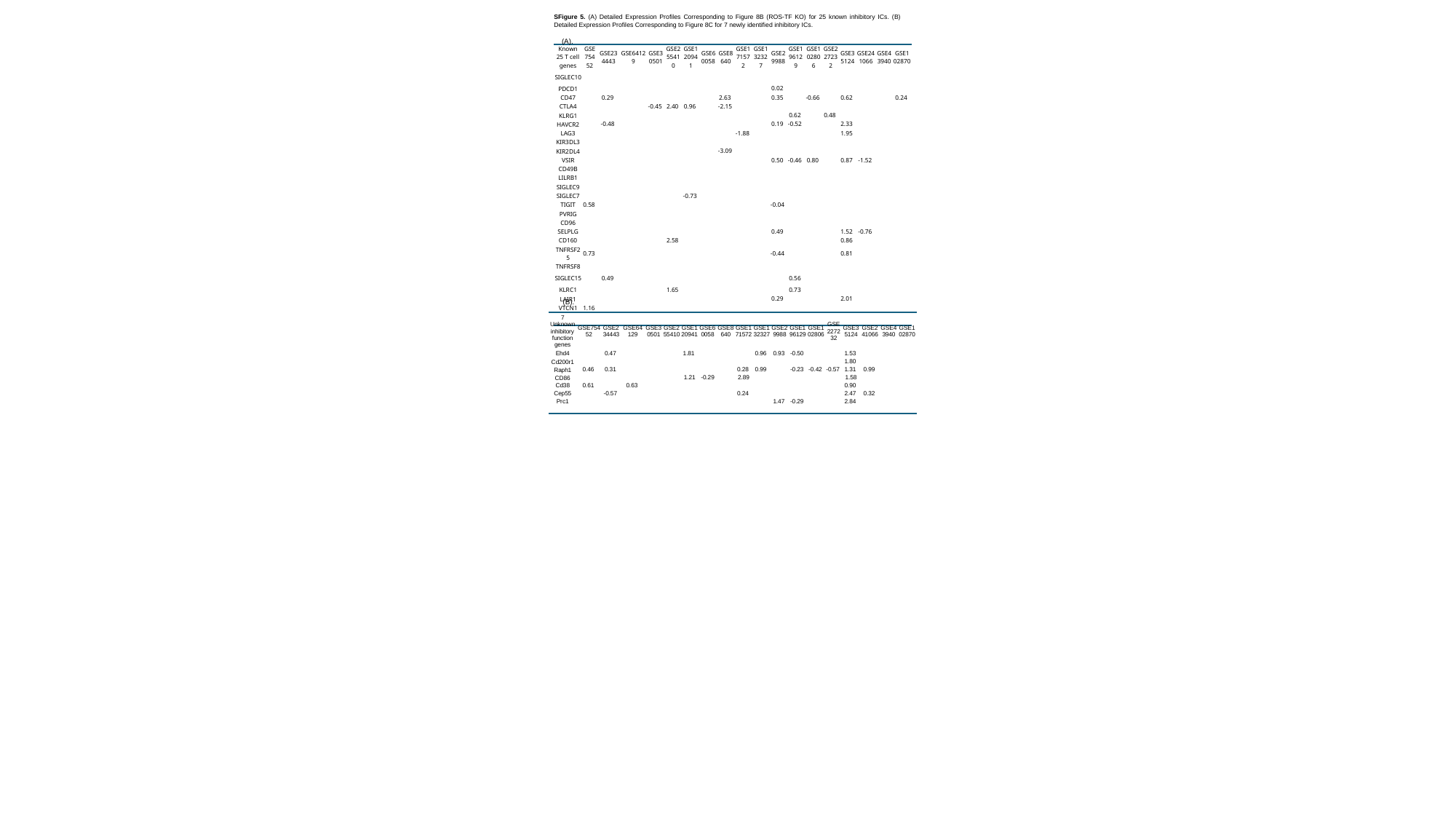

SFigure 5. (A) Detailed Expression Profiles Corresponding to Figure 8B (ROS-TF KO) for 25 known inhibitory ICs. (B) Detailed Expression Profiles Corresponding to Figure 8C for 7 newly identified inhibitory ICs.
(A).
| Known 25 T cell genes | GSE75452 | GSE234443 | GSE64129 | GSE30501 | GSE255410 | GSE120941 | GSE60058 | GSE8640 | GSE171572 | GSE132327 | GSE29988 | GSE196129 | GSE102806 | GSE227232 | GSE35124 | GSE241066 | GSE43940 | GSE102870 |
| --- | --- | --- | --- | --- | --- | --- | --- | --- | --- | --- | --- | --- | --- | --- | --- | --- | --- | --- |
| SIGLEC10 | | | | | | | | | | | | | | | | | | |
| PDCD1 | | | | | | | | | | | 0.02 | | | | | | | |
| CD47 | | 0.29 | | | | | | 2.63 | | | 0.35 | | -0.66 | | 0.62 | | | 0.24 |
| CTLA4 | | | | -0.45 | 2.40 | 0.96 | | -2.15 | | | | | | | | | | |
| KLRG1 | | | | | | | | | | | | 0.62 | | 0.48 | | | | |
| HAVCR2 | | -0.48 | | | | | | | | | 0.19 | -0.52 | | | 2.33 | | | |
| LAG3 | | | | | | | | | -1.88 | | | | | | 1.95 | | | |
| KIR3DL3 | | | | | | | | | | | | | | | | | | |
| KIR2DL4 | | | | | | | | -3.09 | | | | | | | | | | |
| VSIR | | | | | | | | | | | 0.50 | -0.46 | 0.80 | | 0.87 | -1.52 | | |
| CD49B | | | | | | | | | | | | | | | | | | |
| LILRB1 | | | | | | | | | | | | | | | | | | |
| SIGLEC9 | | | | | | | | | | | | | | | | | | |
| SIGLEC7 | | | | | | -0.73 | | | | | | | | | | | | |
| TIGIT | 0.58 | | | | | | | | | | -0.04 | | | | | | | |
| PVRIG | | | | | | | | | | | | | | | | | | |
| CD96 | | | | | | | | | | | | | | | | | | |
| SELPLG | | | | | | | | | | | 0.49 | | | | 1.52 | -0.76 | | |
| CD160 | | | | | 2.58 | | | | | | | | | | 0.86 | | | |
| TNFRSF25 | 0.73 | | | | | | | | | | -0.44 | | | | 0.81 | | | |
| TNFRSF8 | | | | | | | | | | | | | | | | | | |
| SIGLEC15 | | 0.49 | | | | | | | | | | 0.56 | | | | | | |
| KLRC1 | | | | | 1.65 | | | | | | | 0.73 | | | | | | |
| LAIR1 | | | | | | | | | | | 0.29 | | | | 2.01 | | | |
| VTCN1 | 1.16 | | | | | | | | | | | | | | | | | |
| | | | | | | | | | | | | | | | | | | |
(B).
| 7 Unknown inhibitory function genes | GSE75452 | GSE234443 | GSE64129 | GSE30501 | GSE255410 | GSE120941 | GSE60058 | GSE8640 | GSE171572 | GSE132327 | GSE29988 | GSE196129 | GSE102806 | GSE227232 | GSE35124 | GSE241066 | GSE43940 | GSE102870 |
| --- | --- | --- | --- | --- | --- | --- | --- | --- | --- | --- | --- | --- | --- | --- | --- | --- | --- | --- |
| Ehd4 | | 0.47 | | | | 1.81 | | | | 0.96 | 0.93 | -0.50 | | | 1.53 | | | |
| Cd200r1 | | | | | | | | | | | | | | | 1.80 | | | |
| Raph1 | 0.46 | 0.31 | | | | | | | 0.28 | 0.99 | | -0.23 | -0.42 | -0.57 | 1.31 | 0.99 | | |
| CD86 | | | | | | 1.21 | -0.29 | | 2.89 | | | | | | 1.58 | | | |
| Cd38 | 0.61 | | 0.63 | | | | | | | | | | | | 0.90 | | | |
| Cep55 | | -0.57 | | | | | | | 0.24 | | | | | | 2.47 | 0.32 | | |
| Prc1 | | | | | | | | | | | 1.47 | -0.29 | | | 2.84 | | | |
| | | | | | | | | | | | | | | | | | | |

## Slide 6
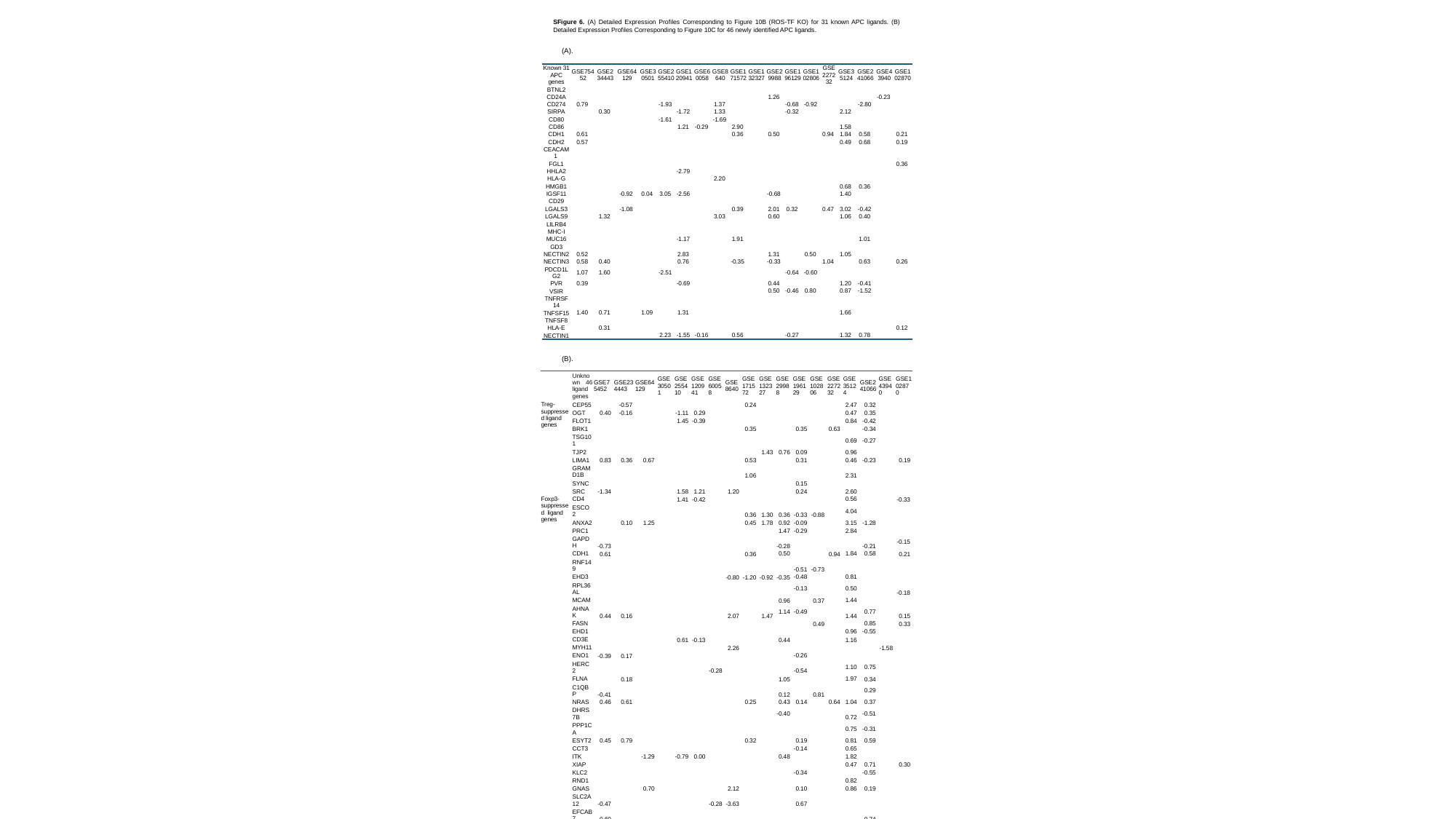

SFigure 6. (A) Detailed Expression Profiles Corresponding to Figure 10B (ROS-TF KO) for 31 known APC ligands. (B) Detailed Expression Profiles Corresponding to Figure 10C for 46 newly identified APC ligands.
(A).
| Known 31 APC genes | GSE75452 | GSE234443 | GSE64129 | GSE30501 | GSE255410 | GSE120941 | GSE60058 | GSE8640 | GSE171572 | GSE132327 | GSE29988 | GSE196129 | GSE102806 | GSE227232 | GSE35124 | GSE241066 | GSE43940 | GSE102870 |
| --- | --- | --- | --- | --- | --- | --- | --- | --- | --- | --- | --- | --- | --- | --- | --- | --- | --- | --- |
| BTNL2 | | | | | | | | | | | | | | | | | | |
| CD24A | | | | | | | | | | | 1.26 | | | | | | -0.23 | |
| CD274 | 0.79 | | | | -1.93 | | | 1.37 | | | | -0.68 | -0.92 | | | -2.80 | | |
| SIRPA | | 0.30 | | | | -1.72 | | 1.33 | | | | -0.32 | | | 2.12 | | | |
| CD80 | | | | | -1.61 | | | -1.69 | | | | | | | | | | |
| CD86 | | | | | | 1.21 | -0.29 | | 2.90 | | | | | | 1.58 | | | |
| CDH1 | 0.61 | | | | | | | | 0.36 | | 0.50 | | | 0.94 | 1.84 | 0.58 | | 0.21 |
| CDH2 | 0.57 | | | | | | | | | | | | | | 0.49 | 0.68 | | 0.19 |
| CEACAM1 | | | | | | | | | | | | | | | | | | |
| FGL1 | | | | | | | | | | | | | | | | | | 0.36 |
| HHLA2 | | | | | | -2.79 | | | | | | | | | | | | |
| HLA-G | | | | | | | | 2.20 | | | | | | | | | | |
| HMGB1 | | | | | | | | | | | | | | | 0.68 | 0.36 | | |
| IGSF11 | | | -0.92 | 0.04 | 3.05 | -2.56 | | | | | -0.68 | | | | 1.40 | | | |
| CD29 | | | | | | | | | | | | | | | | | | |
| LGALS3 | | | -1.08 | | | | | | 0.39 | | 2.01 | 0.32 | | 0.47 | 3.02 | -0.42 | | |
| LGALS9 | | 1.32 | | | | | | 3.03 | | | 0.60 | | | | 1.06 | 0.40 | | |
| LILRB4 | | | | | | | | | | | | | | | | | | |
| MHC-I | | | | | | | | | | | | | | | | | | |
| MUC16 | | | | | | -1.17 | | | 1.91 | | | | | | | 1.01 | | |
| GD3 | | | | | | | | | | | | | | | | | | |
| NECTIN2 | 0.52 | | | | | 2.83 | | | | | 1.31 | | 0.50 | | 1.05 | | | |
| NECTIN3 | 0.58 | 0.40 | | | | 0.76 | | | -0.35 | | -0.33 | | | 1.04 | | 0.63 | | 0.26 |
| PDCD1LG2 | 1.07 | 1.60 | | | -2.51 | | | | | | | -0.64 | -0.60 | | | | | |
| PVR | 0.39 | | | | | -0.69 | | | | | 0.44 | | | | 1.20 | -0.41 | | |
| VSIR | | | | | | | | | | | 0.50 | -0.46 | 0.80 | | 0.87 | -1.52 | | |
| TNFRSF14 | | | | | | | | | | | | | | | | | | |
| TNFSF15 | 1.40 | 0.71 | | 1.09 | | 1.31 | | | | | | | | | 1.66 | | | |
| TNFSF8 | | | | | | | | | | | | | | | | | | |
| HLA-E | | 0.31 | | | | | | | | | | | | | | | | 0.12 |
| NECTIN1 | | | | | 2.23 | -1.55 | -0.16 | | 0.56 | | | -0.27 | | | 1.32 | 0.78 | | |
(B).
| | Unknown 46 ligand genes | GSE75452 | GSE234443 | GSE64129 | GSE30501 | GSE255410 | GSE120941 | GSE60058 | GSE8640 | GSE171572 | GSE132327 | GSE29988 | GSE196129 | GSE102806 | GSE227232 | GSE35124 | GSE241066 | GSE43940 | GSE102870 |
| --- | --- | --- | --- | --- | --- | --- | --- | --- | --- | --- | --- | --- | --- | --- | --- | --- | --- | --- | --- |
| Treg-suppressed ligand genes | CEP55 | | -0.57 | | | | | | | 0.24 | | | | | | 2.47 | 0.32 | | |
| | OGT | 0.40 | -0.16 | | | -1.11 | 0.29 | | | | | | | | | 0.47 | 0.35 | | |
| | FLOT1 | | | | | 1.45 | -0.39 | | | | | | | | | 0.84 | -0.42 | | |
| | BRK1 | | | | | | | | | 0.35 | | | 0.35 | | 0.63 | | -0.34 | | |
| | TSG101 | | | | | | | | | | | | | | | 0.69 | -0.27 | | |
| | TJP2 | | | | | | | | | | 1.43 | 0.76 | 0.09 | | | 0.96 | | | |
| | LIMA1 | 0.83 | 0.36 | 0.67 | | | | | | 0.53 | | | 0.31 | | | 0.46 | -0.23 | | 0.19 |
| | GRAMD1B | | | | | | | | | 1.06 | | | | | | 2.31 | | | |
| | SYNC | | | | | | | | | | | | 0.15 | | | | | | |
| | SRC | -1.34 | | | | 1.58 | 1.21 | | 1.20 | | | | 0.24 | | | 2.60 | | | |
| Foxp3-suppressed ligand genes | CD4 | | | | | 1.41 | -0.42 | | | | | | | | | 0.56 | | | -0.33 |
| | ESCO2 | | | | | | | | | 0.36 | 1.30 | 0.36 | -0.33 | -0.88 | | 4.04 | | | |
| | ANXA2 | | 0.10 | 1.25 | | | | | | 0.45 | 1.78 | 0.92 | -0.09 | | | 3.15 | -1.28 | | |
| | PRC1 | | | | | | | | | | | 1.47 | -0.29 | | | 2.84 | | | |
| | GAPDH | -0.73 | | | | | | | | | | -0.28 | | | | | -0.21 | | -0.15 |
| | CDH1 | 0.61 | | | | | | | | 0.36 | | 0.50 | | | 0.94 | 1.84 | 0.58 | | 0.21 |
| | RNF149 | | | | | | | | | | | | -0.51 | -0.73 | | | | | |
| | EHD3 | | | | | | | | -0.80 | -1.20 | -0.92 | -0.35 | -0.48 | | | 0.81 | | | |
| | RPL36AL | | | | | | | | | | | | -0.13 | | | 0.50 | | | -0.18 |
| | MCAM | | | | | | | | | | | 0.96 | | 0.37 | | 1.44 | | | |
| | AHNAK | 0.44 | 0.16 | | | | | | 2.07 | | 1.47 | 1.14 | -0.49 | | | 1.44 | 0.77 | | 0.15 |
| | FASN | | | | | | | | | | | | | 0.49 | | | 0.85 | | 0.33 |
| | EHD1 | | | | | | | | | | | | | | | 0.96 | -0.55 | | |
| | CD3E | | | | | 0.61 | -0.13 | | | | | 0.44 | | | | 1.16 | | | |
| | MYH11 | | | | | | | | 2.26 | | | | | | | | | -1.58 | |
| | ENO1 | -0.39 | 0.17 | | | | | | | | | | -0.26 | | | | | | |
| | HERC2 | | | | | | | -0.28 | | | | | -0.54 | | | 1.10 | 0.75 | | |
| | FLNA | | 0.18 | | | | | | | | | 1.05 | | | | 1.97 | 0.34 | | |
| | C1QBP | -0.41 | | | | | | | | | | 0.12 | | 0.81 | | | 0.29 | | |
| | NRAS | 0.46 | 0.61 | | | | | | | 0.25 | | 0.43 | 0.14 | | 0.64 | 1.04 | 0.37 | | |
| | DHRS7B | | | | | | | | | | | -0.40 | | | | 0.72 | -0.51 | | |
| | PPP1CA | | | | | | | | | | | | | | | 0.75 | -0.31 | | |
| | ESYT2 | 0.45 | 0.79 | | | | | | | 0.32 | | | 0.19 | | | 0.81 | 0.59 | | |
| | CCT3 | | | | | | | | | | | | -0.14 | | | 0.65 | | | |
| | ITK | | | -1.29 | | -0.79 | 0.00 | | | | | 0.48 | | | | 1.82 | | | |
| | XIAP | | | | | | | | | | | | | | | 0.47 | 0.71 | | 0.30 |
| | KLC2 | | | | | | | | | | | | -0.34 | | | | -0.55 | | |
| | RND1 | | | | | | | | | | | | | | | 0.82 | | | |
| | GNAS | | | 0.70 | | | | | 2.12 | | | | 0.10 | | | 0.86 | 0.19 | | |
| | SLC2A12 | -0.47 | | | | | | -0.28 | -3.63 | | | | 0.67 | | | | | | |
| | EFCAB7 | -0.60 | | | | | | | | | | | | | | | 0.74 | | |
| | WDR1 | 0.51 | 0.69 | | | | | | | | | 0.47 | | | | 0.85 | -0.17 | | |
| | SHANK2 | | | | | | | | | | | | -0.72 | | 0.71 | | | | |
| | CTTN | | | | | | | | | | | | 0.16 | 0.59 | 1.29 | 1.90 | 0.15 | | -0.12 |
| | PCDH1 | | | | | | | | | | | | | | | 0.74 | | | |
| | PVR | 0.39 | | | | | -0.69 | | | | | 0.44 | | | | 1.12 | 0.41 | | |

## Slide 7
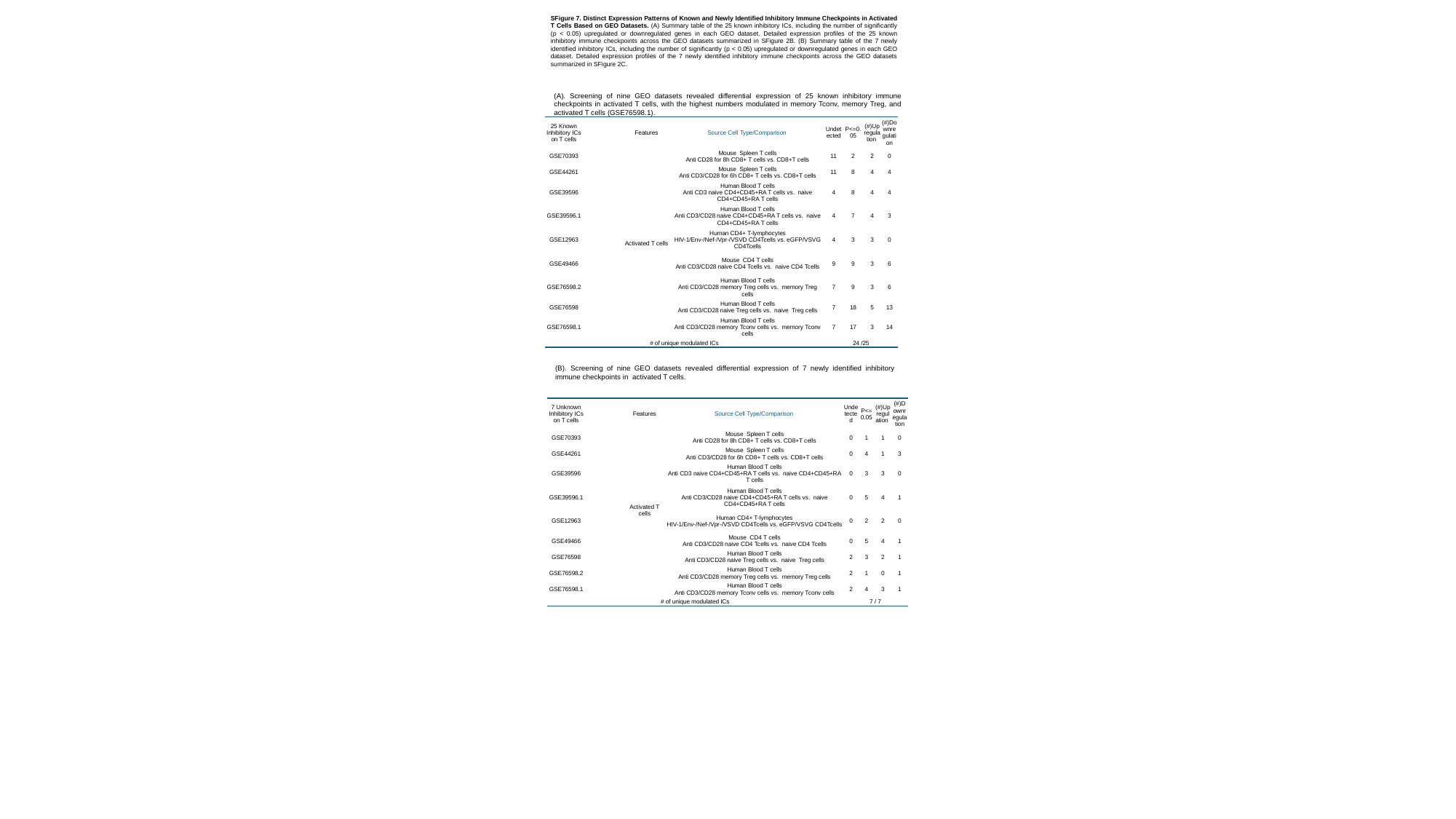

SFigure 7. Distinct Expression Patterns of Known and Newly Identified Inhibitory Immune Checkpoints in Activated T Cells Based on GEO Datasets. (A) Summary table of the 25 known inhibitory ICs, including the number of significantly (p < 0.05) upregulated or downregulated genes in each GEO dataset. Detailed expression profiles of the 25 known inhibitory immune checkpoints across the GEO datasets summarized in SFigure 2B. (B) Summary table of the 7 newly identified inhibitory ICs, including the number of significantly (p < 0.05) upregulated or downregulated genes in each GEO dataset. Detailed expression profiles of the 7 newly identified inhibitory immune checkpoints across the GEO datasets summarized in SFigure 2C.
(A). Screening of nine GEO datasets revealed differential expression of 25 known inhibitory immune checkpoints in activated T cells, with the highest numbers modulated in memory Tconv, memory Treg, and activated T cells (GSE76598.1).
| 25 Known Inhibitory ICs on T cells | | Features | Source Cell Type/Comparison | Undetected | P<=0.05 | (#)Upregulation | (#)Downregulation |
| --- | --- | --- | --- | --- | --- | --- | --- |
| GSE70393 | | Activated T cells | Mouse Spleen T cells Anti CD28 for 8h CD8+ T cells vs. CD8+T cells | 11 | 2 | 2 | 0 |
| GSE44261 | | | Mouse Spleen T cells Anti CD3/CD28 for 6h CD8+ T cells vs. CD8+T cells | 11 | 8 | 4 | 4 |
| GSE39596 | | | Human Blood T cells Anti CD3 naive CD4+CD45+RA T cells vs. naive CD4+CD45+RA T cells | 4 | 8 | 4 | 4 |
| GSE39596.1 | | | Human Blood T cells Anti CD3/CD28 naive CD4+CD45+RA T cells vs. naive CD4+CD45+RA T cells | 4 | 7 | 4 | 3 |
| GSE12963 | | | Human CD4+ T-lymphocytes HIV-1/Env-/Nef-/Vpr-/VSVD CD4Tcells vs. eGFP/VSVG CD4Tcells | 4 | 3 | 3 | 0 |
| GSE49466 | | | Mouse CD4 T cells Anti CD3/CD28 naive CD4 Tcells vs. naive CD4 Tcells | 9 | 9 | 3 | 6 |
| GSE76598.2 | | | Human Blood T cells Anti CD3/CD28 memory Treg cells vs. memory Treg cells | 7 | 9 | 3 | 6 |
| GSE76598 | | | Human Blood T cells Anti CD3/CD28 naive Treg cells vs. naive Treg cells | 7 | 18 | 5 | 13 |
| GSE76598.1 | | | Human Blood T cells Anti CD3/CD28 memory Tconv cells vs. memory Tconv cells | 7 | 17 | 3 | 14 |
| # of unique modulated ICs | | | | 24 /25 | | | |
(B). Screening of nine GEO datasets revealed differential expression of 7 newly identified inhibitory immune checkpoints in activated T cells.
| 7 Unknown Inhibitory ICs on T cells | | Features | Source Cell Type/Comparison | Undetected | P<=0.05 | (#)Upregulation | (#)Downregulation |
| --- | --- | --- | --- | --- | --- | --- | --- |
| GSE70393 | | Activated T cells | Mouse Spleen T cells Anti CD28 for 8h CD8+ T cells vs. CD8+T cells | 0 | 1 | 1 | 0 |
| GSE44261 | | | Mouse Spleen T cells Anti CD3/CD28 for 6h CD8+ T cells vs. CD8+T cells | 0 | 4 | 1 | 3 |
| GSE39596 | | | Human Blood T cells Anti CD3 naive CD4+CD45+RA T cells vs. naive CD4+CD45+RA T cells | 0 | 3 | 3 | 0 |
| GSE39596.1 | | | Human Blood T cells Anti CD3/CD28 naive CD4+CD45+RA T cells vs. naive CD4+CD45+RA T cells | 0 | 5 | 4 | 1 |
| GSE12963 | | | Human CD4+ T-lymphocytes HIV-1/Env-/Nef-/Vpr-/VSVD CD4Tcells vs. eGFP/VSVG CD4Tcells | 0 | 2 | 2 | 0 |
| GSE49466 | | | Mouse CD4 T cells Anti CD3/CD28 naive CD4 Tcells vs. naive CD4 Tcells | 0 | 5 | 4 | 1 |
| GSE76598 | | | Human Blood T cells Anti CD3/CD28 naive Treg cells vs. naive Treg cells | 2 | 3 | 2 | 1 |
| GSE76598.2 | | | Human Blood T cells Anti CD3/CD28 memory Treg cells vs. memory Treg cells | 2 | 1 | 0 | 1 |
| GSE76598.1 | | | Human Blood T cells Anti CD3/CD28 memory Tconv cells vs. memory Tconv cells | 2 | 4 | 3 | 1 |
| # of unique modulated ICs | | | | 7 / 7 | | | |

## Slide 8
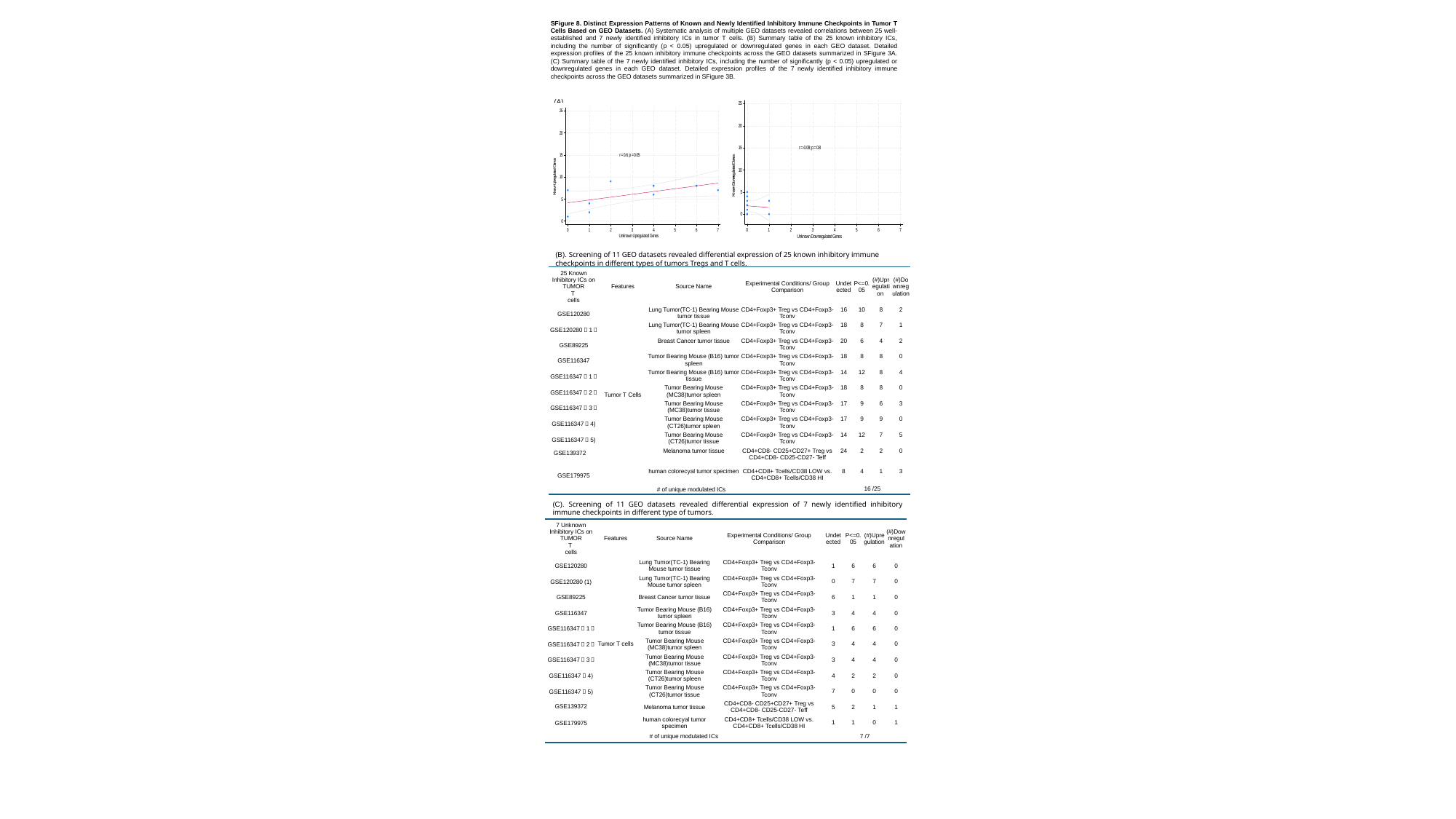

SFigure 8. Distinct Expression Patterns of Known and Newly Identified Inhibitory Immune Checkpoints in Tumor T Cells Based on GEO Datasets. (A) Systematic analysis of multiple GEO datasets revealed correlations between 25 well-established and 7 newly identified inhibitory ICs in tumor T cells. (B) Summary table of the 25 known inhibitory ICs, including the number of significantly (p < 0.05) upregulated or downregulated genes in each GEO dataset. Detailed expression profiles of the 25 known inhibitory immune checkpoints across the GEO datasets summarized in SFigure 3A. (C) Summary table of the 7 newly identified inhibitory ICs, including the number of significantly (p < 0.05) upregulated or downregulated genes in each GEO dataset. Detailed expression profiles of the 7 newly identified inhibitory immune checkpoints across the GEO datasets summarized in SFigure 3B.
(A).
(B). Screening of 11 GEO datasets revealed differential expression of 25 known inhibitory immune checkpoints in different types of tumors Tregs and T cells.
| 25 Known Inhibitory ICs on TUMOR T cells | Features | Source Name | Experimental Conditions/ Group Comparison | Undetected | P<=0.05 | (#)Upregulation | (#)Downregulation |
| --- | --- | --- | --- | --- | --- | --- | --- |
| GSE120280 | Tumor T Cells | Lung Tumor(TC-1) Bearing Mouse tumor tissue | CD4+Foxp3+ Treg vs CD4+Foxp3- Tconv | 16 | 10 | 8 | 2 |
| GSE120280（1） | | Lung Tumor(TC-1) Bearing Mouse tumor spleen | CD4+Foxp3+ Treg vs CD4+Foxp3- Tconv | 18 | 8 | 7 | 1 |
| GSE89225 | | Breast Cancer tumor tissue | CD4+Foxp3+ Treg vs CD4+Foxp3- Tconv | 20 | 6 | 4 | 2 |
| GSE116347 | | Tumor Bearing Mouse (B16) tumor spleen | CD4+Foxp3+ Treg vs CD4+Foxp3- Tconv | 18 | 8 | 8 | 0 |
| GSE116347（1） | | Tumor Bearing Mouse (B16) tumor tissue | CD4+Foxp3+ Treg vs CD4+Foxp3- Tconv | 14 | 12 | 8 | 4 |
| GSE116347（2） | | Tumor Bearing Mouse (MC38)tumor spleen | CD4+Foxp3+ Treg vs CD4+Foxp3- Tconv | 18 | 8 | 8 | 0 |
| GSE116347（3） | | Tumor Bearing Mouse (MC38)tumor tissue | CD4+Foxp3+ Treg vs CD4+Foxp3- Tconv | 17 | 9 | 6 | 3 |
| GSE116347（4) | | Tumor Bearing Mouse (CT26)tumor spleen | CD4+Foxp3+ Treg vs CD4+Foxp3- Tconv | 17 | 9 | 9 | 0 |
| GSE116347（5) | | Tumor Bearing Mouse (CT26)tumor tissue | CD4+Foxp3+ Treg vs CD4+Foxp3- Tconv | 14 | 12 | 7 | 5 |
| GSE139372 | | Melanoma tumor tissue | CD4+CD8- CD25+CD27+ Treg vs CD4+CD8- CD25-CD27- Teff | 24 | 2 | 2 | 0 |
| GSE179975 | | human colorecyal tumor specimen | CD4+CD8+ Tcells/CD38 LOW vs. CD4+CD8+ Tcells/CD38 HI | 8 | 4 | 1 | 3 |
| # of unique modulated ICs | | | | 16 /25 | | | |
(C). Screening of 11 GEO datasets revealed differential expression of 7 newly identified inhibitory immune checkpoints in different type of tumors.
| 7 Unknown Inhibitory ICs on TUMOR T cells | Features | Source Name | Experimental Conditions/ Group Comparison | Undetected | P<=0.05 | (#)Upregulation | (#)Downregulation |
| --- | --- | --- | --- | --- | --- | --- | --- |
| GSE120280 | Tumor T cells | Lung Tumor(TC-1) Bearing Mouse tumor tissue | CD4+Foxp3+ Treg vs CD4+Foxp3- Tconv | 1 | 6 | 6 | 0 |
| GSE120280 (1) | | Lung Tumor(TC-1) Bearing Mouse tumor spleen | CD4+Foxp3+ Treg vs CD4+Foxp3- Tconv | 0 | 7 | 7 | 0 |
| GSE89225 | | Breast Cancer tumor tissue | CD4+Foxp3+ Treg vs CD4+Foxp3- Tconv | 6 | 1 | 1 | 0 |
| GSE116347 | | Tumor Bearing Mouse (B16) tumor spleen | CD4+Foxp3+ Treg vs CD4+Foxp3- Tconv | 3 | 4 | 4 | 0 |
| GSE116347（1） | | Tumor Bearing Mouse (B16) tumor tissue | CD4+Foxp3+ Treg vs CD4+Foxp3- Tconv | 1 | 6 | 6 | 0 |
| GSE116347（2） | | Tumor Bearing Mouse (MC38)tumor spleen | CD4+Foxp3+ Treg vs CD4+Foxp3- Tconv | 3 | 4 | 4 | 0 |
| GSE116347（3） | | Tumor Bearing Mouse (MC38)tumor tissue | CD4+Foxp3+ Treg vs CD4+Foxp3- Tconv | 3 | 4 | 4 | 0 |
| GSE116347（4) | | Tumor Bearing Mouse (CT26)tumor spleen | CD4+Foxp3+ Treg vs CD4+Foxp3- Tconv | 4 | 2 | 2 | 0 |
| GSE116347（5) | | Tumor Bearing Mouse (CT26)tumor tissue | CD4+Foxp3+ Treg vs CD4+Foxp3- Tconv | 7 | 0 | 0 | 0 |
| GSE139372 | | Melanoma tumor tissue | CD4+CD8- CD25+CD27+ Treg vs CD4+CD8- CD25-CD27- Teff | 5 | 2 | 1 | 1 |
| GSE179975 | | human colorecyal tumor specimen | CD4+CD8+ Tcells/CD38 LOW vs. CD4+CD8+ Tcells/CD38 HI | 1 | 1 | 0 | 1 |
| # of unique modulated ICs | | | | 7 /7 | | | |

## Slide 9
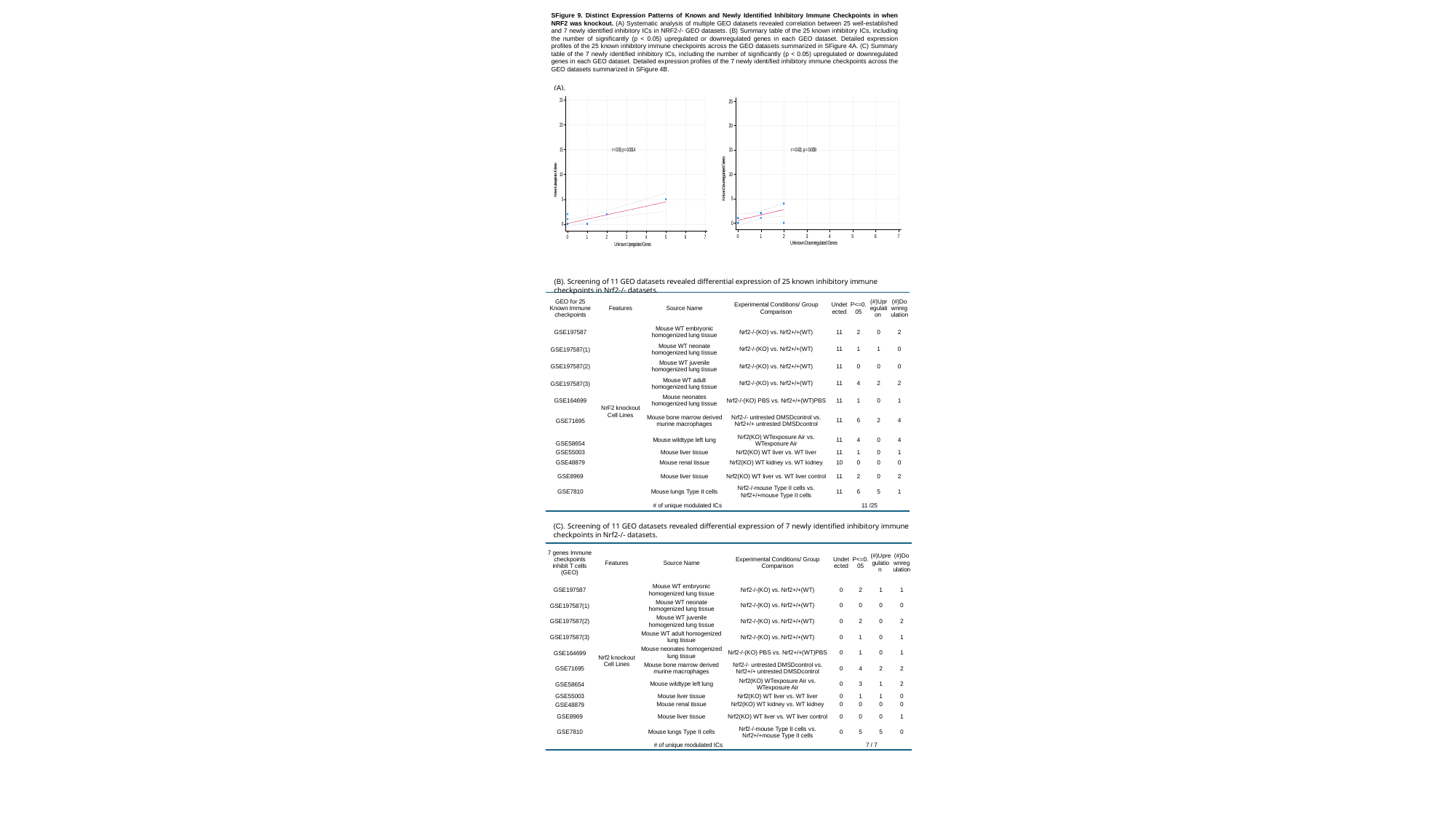

SFigure 9. Distinct Expression Patterns of Known and Newly Identified Inhibitory Immune Checkpoints in when NRF2 was knockout. (A) Systematic analysis of multiple GEO datasets revealed correlation between 25 well-established and 7 newly identified inhibitory ICs in NRF2-/- GEO datasets. (B) Summary table of the 25 known inhibitory ICs, including the number of significantly (p < 0.05) upregulated or downregulated genes in each GEO dataset. Detailed expression profiles of the 25 known inhibitory immune checkpoints across the GEO datasets summarized in SFigure 4A. (C) Summary table of the 7 newly identified inhibitory ICs, including the number of significantly (p < 0.05) upregulated or downregulated genes in each GEO dataset. Detailed expression profiles of the 7 newly identified inhibitory immune checkpoints across the GEO datasets summarized in SFigure 4B.
(A).
(B). Screening of 11 GEO datasets revealed differential expression of 25 known inhibitory immune checkpoints in Nrf2-/- datasets.
| GEO for 25 Known Immune checkpoints | Features | Source Name | Experimental Conditions/ Group Comparison | Undetected | P<=0.05 | (#)Upregulation | (#)Downregulation |
| --- | --- | --- | --- | --- | --- | --- | --- |
| GSE197587 | NrF2 knockout Cell Lines | Mouse WT embryonic homogenized lung tissue | Nrf2-/-(KO) vs. Nrf2+/+(WT) | 11 | 2 | 0 | 2 |
| GSE197587(1) | | Mouse WT neonate homogenized lung tissue | Nrf2-/-(KO) vs. Nrf2+/+(WT) | 11 | 1 | 1 | 0 |
| GSE197587(2) | | Mouse WT juvenile homogenized lung tissue | Nrf2-/-(KO) vs. Nrf2+/+(WT) | 11 | 0 | 0 | 0 |
| GSE197587(3) | | Mouse WT adult homogenized lung tissue | Nrf2-/-(KO) vs. Nrf2+/+(WT) | 11 | 4 | 2 | 2 |
| GSE164699 | | Mouse neonates homogenized lung tissue | Nrf2-/-(KO) PBS vs. Nrf2+/+(WT)PBS | 11 | 1 | 0 | 1 |
| GSE71695 | | Mouse bone marrow derived murine macrophages | Nrf2-/- untrested DMSDcontrol vs. Nrf2+/+ untrested DMSDcontrol | 11 | 6 | 2 | 4 |
| GSE58654 GSE58654 | | Mouse wildtype left lung | Nrf2(KO) WTexposure Air vs. WTexposure Air | 11 | 4 | 0 | 4 |
| GSE55003 | | Mouse liver tissue | Nrf2(KO) WT liver vs. WT liver | 11 | 1 | 0 | 1 |
| GSE48879 | | Mouse renal tissue | Nrf2(KO) WT kidney vs. WT kidney | 10 | 0 | 0 | 0 |
| GSE8969 | | Mouse liver tissue | Nrf2(KO) WT liver vs. WT liver control | 11 | 2 | 0 | 2 |
| GSE7810 | | Mouse lungs Type II cells | Nrf2-/-mouse Type II cells vs. Nrf2+/+mouse Type II cells | 11 | 6 | 5 | 1 |
| # of unique modulated ICs | | | | 11 /25 | | | |
(C). Screening of 11 GEO datasets revealed differential expression of 7 newly identified inhibitory immune checkpoints in Nrf2-/- datasets.
| 7 genes Immune checkpoints inhibit T cells (GEO) | Features | Source Name | Experimental Conditions/ Group Comparison | Undetected | P<=0.05 | (#)Upregulation | (#)Downregulation |
| --- | --- | --- | --- | --- | --- | --- | --- |
| GSE197587 | Nrf2 knockout Cell Lines | Mouse WT embryonic homogenized lung tissue | Nrf2-/-(KO) vs. Nrf2+/+(WT) | 0 | 2 | 1 | 1 |
| GSE197587(1) | | Mouse WT neonate homogenized lung tissue | Nrf2-/-(KO) vs. Nrf2+/+(WT) | 0 | 0 | 0 | 0 |
| GSE197587(2) | | Mouse WT juvenile homogenized lung tissue | Nrf2-/-(KO) vs. Nrf2+/+(WT) | 0 | 2 | 0 | 2 |
| GSE197587(3) | | Mouse WT adult homogenized lung tissue | Nrf2-/-(KO) vs. Nrf2+/+(WT) | 0 | 1 | 0 | 1 |
| GSE164699 | | Mouse neonates homogenized lung tissue | Nrf2-/-(KO) PBS vs. Nrf2+/+(WT)PBS | 0 | 1 | 0 | 1 |
| GSE71695 | | Mouse bone marrow derived murine macrophages | Nrf2-/- untrested DMSDcontrol vs. Nrf2+/+ untrested DMSDcontrol | 0 | 4 | 2 | 2 |
| GSE58654 | | Mouse wildtype left lung | Nrf2(KO) WTexposure Air vs. WTexposure Air | 0 | 3 | 1 | 2 |
| GSE55003 | | Mouse liver tissue | Nrf2(KO) WT liver vs. WT liver | 0 | 1 | 1 | 0 |
| GSE48879 | | Mouse renal tissue | Nrf2(KO) WT kidney vs. WT kidney | 0 | 0 | 0 | 0 |
| GSE8969 | | Mouse liver tissue | Nrf2(KO) WT liver vs. WT liver control | 0 | 0 | 0 | 1 |
| GSE7810 | | Mouse lungs Type II cells | Nrf2-/-mouse Type II cells vs. Nrf2+/+mouse Type II cells | 0 | 5 | 5 | 0 |
| # of unique modulated ICs | | | | 7 / 7 | | | |

## Slide 10
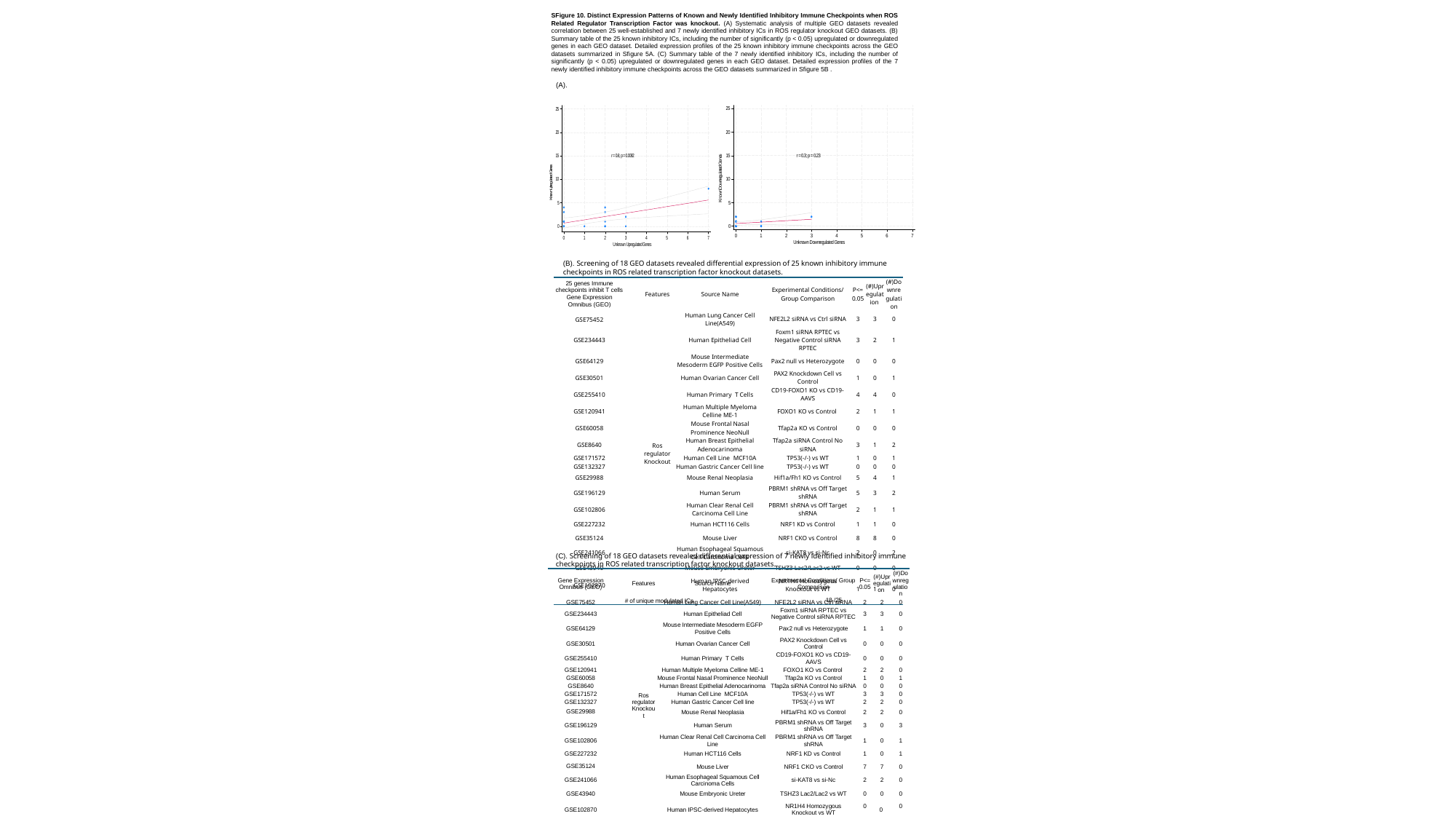

SFigure 10. Distinct Expression Patterns of Known and Newly Identified Inhibitory Immune Checkpoints when ROS Related Regulator Transcription Factor was knockout. (A) Systematic analysis of multiple GEO datasets revealed correlation between 25 well-established and 7 newly identified inhibitory ICs in ROS regulator knockout GEO datasets. (B) Summary table of the 25 known inhibitory ICs, including the number of significantly (p < 0.05) upregulated or downregulated genes in each GEO dataset. Detailed expression profiles of the 25 known inhibitory immune checkpoints across the GEO datasets summarized in Sfigure 5A. (C) Summary table of the 7 newly identified inhibitory ICs, including the number of significantly (p < 0.05) upregulated or downregulated genes in each GEO dataset. Detailed expression profiles of the 7 newly identified inhibitory immune checkpoints across the GEO datasets summarized in Sfigure 5B .
(A).
(B). Screening of 18 GEO datasets revealed differential expression of 25 known inhibitory immune checkpoints in ROS related transcription factor knockout datasets.
| 25 genes Immune checkpoints inhibit T cells Gene Expression Omnibus (GEO) | | Features | Source Name | Experimental Conditions/ Group Comparison | P<=0.05 | (#)Upregulation | (#)Downregulation |
| --- | --- | --- | --- | --- | --- | --- | --- |
| GSE75452 | | Ros regulator Knockout | Human Lung Cancer Cell Line(A549) | NFE2L2 siRNA vs Ctrl siRNA | 3 | 3 | 0 |
| GSE234443 | | | Human Epitheliad Cell | Foxm1 siRNA RPTEC vs Negative Control siRNA RPTEC | 3 | 2 | 1 |
| GSE64129 | | | Mouse Intermediate Mesoderm EGFP Positive Cells | Pax2 null vs Heterozygote | 0 | 0 | 0 |
| GSE30501 | | | Human Ovarian Cancer Cell | PAX2 Knockdown Cell vs Control | 1 | 0 | 1 |
| GSE255410 | | | Human Primary T Cells | CD19-FOXO1 KO vs CD19-AAVS | 4 | 4 | 0 |
| GSE120941 | | | Human Multiple Myeloma Celline ME-1 | FOXO1 KO vs Control | 2 | 1 | 1 |
| GSE60058 | | | Mouse Frontal Nasal Prominence NeoNull | Tfap2a KO vs Control | 0 | 0 | 0 |
| GSE8640 | | | Human Breast Epithelial Adenocarinoma | Tfap2a siRNA Control No siRNA | 3 | 1 | 2 |
| GSE171572 | | | Human Cell Line MCF10A | TP53(-/-) vs WT | 1 | 0 | 1 |
| GSE132327 | | | Human Gastric Cancer Cell line | TP53(-/-) vs WT | 0 | 0 | 0 |
| GSE29988 | | | Mouse Renal Neoplasia | Hif1a/Fh1 KO vs Control | 5 | 4 | 1 |
| GSE196129 | | | Human Serum | PBRM1 shRNA vs Off Target shRNA | 5 | 3 | 2 |
| GSE102806 | | | Human Clear Renal Cell Carcinoma Cell Line | PBRM1 shRNA vs Off Target shRNA | 2 | 1 | 1 |
| GSE227232 | | | Human HCT116 Cells | NRF1 KD vs Control | 1 | 1 | 0 |
| GSE35124 | | | Mouse Liver | NRF1 CKO vs Control | 8 | 8 | 0 |
| GSE241066 | | | Human Esophageal Squamous Cell Carcinoma Cells | si-KAT8 vs si-Nc | 2 | 0 | 2 |
| GSE43940 | | | Mouse Embryonic Ureter | TSHZ3 Lac2/Lac2 vs WT | 0 | 0 | 0 |
| GSE102870 | | | Human IPSC-derived Hepatocytes | NR1H4 Homozygous Knockout vs WT | 1 | 1 | 0 |
| # of unique modulated ICs | | | | 18 /25 | | | |
(C). Screening of 18 GEO datasets revealed differential expression of 7 newly identified inhibitory immune checkpoints in ROS related transcription factor knockout datasets.
| Gene Expression Omnibus (GEO) | | Features | Source Name | Experimental Conditions/ Group Comparison | P<=0.05 | (#)Upregulation | (#)Downregulation |
| --- | --- | --- | --- | --- | --- | --- | --- |
| GSE75452 | | Ros regulator Knockout | Human Lung Cancer Cell Line(A549) | NFE2L2 siRNA vs Ctrl siRNA | 2 | 2 | 0 |
| GSE234443 | | | Human Epitheliad Cell | Foxm1 siRNA RPTEC vs Negative Control siRNA RPTEC | 3 | 3 | 0 |
| GSE64129 | | | Mouse Intermediate Mesoderm EGFP Positive Cells | Pax2 null vs Heterozygote | 1 | 1 | 0 |
| GSE30501 | | | Human Ovarian Cancer Cell | PAX2 Knockdown Cell vs Control | 0 | 0 | 0 |
| GSE255410 | | | Human Primary T Cells | CD19-FOXO1 KO vs CD19-AAVS | 0 | 0 | 0 |
| GSE120941 | | | Human Multiple Myeloma Celline ME-1 | FOXO1 KO vs Control | 2 | 2 | 0 |
| GSE60058 | | | Mouse Frontal Nasal Prominence NeoNull | Tfap2a KO vs Control | 1 | 0 | 1 |
| GSE8640 | | | Human Breast Epithelial Adenocarinoma | Tfap2a siRNA Control No siRNA | 0 | 0 | 0 |
| GSE171572 | | | Human Cell Line MCF10A | TP53(-/-) vs WT | 3 | 3 | 0 |
| GSE132327 | | | Human Gastric Cancer Cell line | TP53(-/-) vs WT | 2 | 2 | 0 |
| GSE29988 | | | Mouse Renal Neoplasia | Hif1a/Fh1 KO vs Control | 2 | 2 | 0 |
| GSE196129 | | | Human Serum | PBRM1 shRNA vs Off Target shRNA | 3 | 0 | 3 |
| GSE102806 | | | Human Clear Renal Cell Carcinoma Cell Line | PBRM1 shRNA vs Off Target shRNA | 1 | 0 | 1 |
| GSE227232 | | | Human HCT116 Cells | NRF1 KD vs Control | 1 | 0 | 1 |
| GSE35124 | | | Mouse Liver | NRF1 CKO vs Control | 7 | 7 | 0 |
| GSE241066 | | | Human Esophageal Squamous Cell Carcinoma Cells | si-KAT8 vs si-Nc | 2 | 2 | 0 |
| GSE43940 | | | Mouse Embryonic Ureter | TSHZ3 Lac2/Lac2 vs WT | 0 | 0 | 0 |
| GSE102870 | | | Human IPSC-derived Hepatocytes | NR1H4 Homozygous Knockout vs WT | 0 | 0 | 0 |
| # of unique modulated ICs | | | | 7 / 7 | | | |

## Slide 11
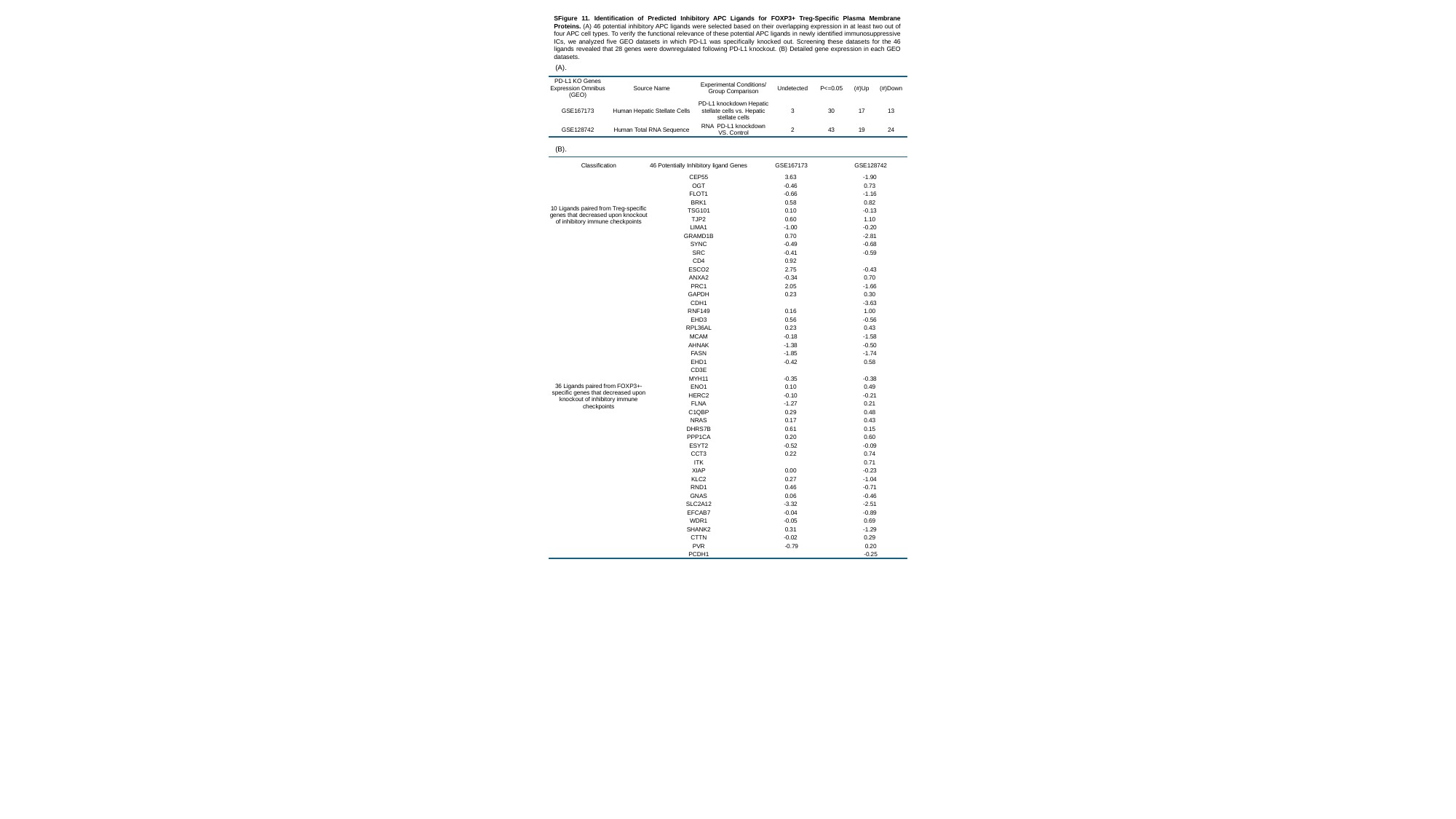

SFigure 11. Identification of Predicted Inhibitory APC Ligands for FOXP3+ Treg-Specific Plasma Membrane Proteins. (A) 46 potential inhibitory APC ligands were selected based on their overlapping expression in at least two out of four APC cell types. To verify the functional relevance of these potential APC ligands in newly identified immunosuppressive ICs, we analyzed five GEO datasets in which PD-L1 was specifically knocked out. Screening these datasets for the 46 ligands revealed that 28 genes were downregulated following PD-L1 knockout. (B) Detailed gene expression in each GEO datasets.
(A).
| PD-L1 KO Genes Expression Omnibus (GEO) | Source Name | Experimental Conditions/ Group Comparison | Undetected | P<=0.05 | (#)Up | (#)Down |
| --- | --- | --- | --- | --- | --- | --- |
| GSE167173 | Human Hepatic Stellate Cells | PD-L1 knockdown Hepatic stellate cells vs. Hepatic stellate cells | 3 | 30 | 17 | 13 |
| GSE128742 | Human Total RNA Sequence | RNA PD-L1 knockdown VS. Control | 2 | 43 | 19 | 24 |
(B).
| Classification | 46 Potentially Inhibitory ligand Genes | GSE167173 | GSE128742 |
| --- | --- | --- | --- |
| 10 Ligands paired from Treg-specific genes that decreased upon knockout of inhibitory immune checkpoints | CEP55 | 3.63 | -1.90 |
| | OGT | -0.46 | 0.73 |
| | FLOT1 | -0.66 | -1.16 |
| | BRK1 | 0.58 | 0.82 |
| | TSG101 | 0.10 | -0.13 |
| | TJP2 | 0.60 | 1.10 |
| | LIMA1 | -1.00 | -0.20 |
| | GRAMD1B | 0.70 | -2.81 |
| | SYNC | -0.49 | -0.68 |
| | SRC | -0.41 | -0.59 |
| 36 Ligands paired from FOXP3+-specific genes that decreased upon knockout of inhibitory immune checkpoints | CD4 | 0.92 | |
| | ESCO2 | 2.75 | -0.43 |
| | ANXA2 | -0.34 | 0.70 |
| | PRC1 | 2.05 | -1.66 |
| | GAPDH | 0.23 | 0.30 |
| | CDH1 | | -3.63 |
| | RNF149 | 0.16 | 1.00 |
| | EHD3 | 0.56 | -0.56 |
| | RPL36AL | 0.23 | 0.43 |
| | MCAM | -0.18 | -1.58 |
| | AHNAK | -1.38 | -0.50 |
| | FASN | -1.85 | -1.74 |
| | EHD1 | -0.42 | 0.58 |
| | CD3E | | |
| | MYH11 | -0.35 | -0.38 |
| | ENO1 | 0.10 | 0.49 |
| | HERC2 | -0.10 | -0.21 |
| | FLNA | -1.27 | 0.21 |
| | C1QBP | 0.29 | 0.48 |
| | NRAS | 0.17 | 0.43 |
| | DHRS7B | 0.61 | 0.15 |
| | PPP1CA | 0.20 | 0.60 |
| | ESYT2 | -0.52 | -0.09 |
| | CCT3 | 0.22 | 0.74 |
| | ITK | | 0.71 |
| | XIAP | 0.00 | -0.23 |
| | KLC2 | 0.27 | -1.04 |
| | RND1 | 0.46 | -0.71 |
| | GNAS | 0.06 | -0.46 |
| | SLC2A12 | -3.32 | -2.51 |
| | EFCAB7 | -0.04 | -0.89 |
| | WDR1 | -0.05 | 0.69 |
| | SHANK2 | 0.31 | -1.29 |
| | CTTN | -0.02 | 0.29 |
| | PVR | -0.79 | 0.20 |
| | PCDH1 | | -0.25 |

## Slide 12
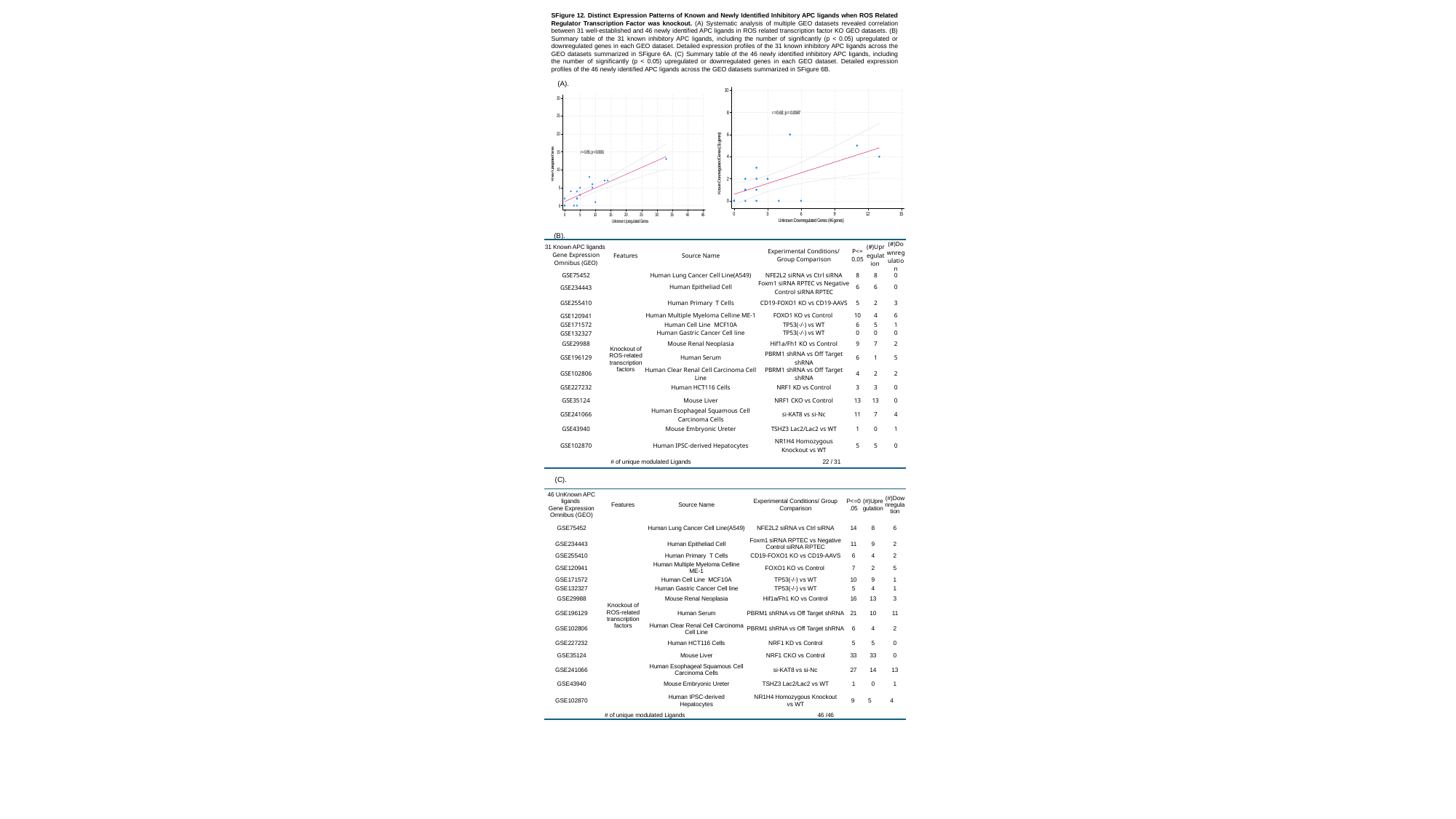

SFigure 12. Distinct Expression Patterns of Known and Newly Identified Inhibitory APC ligands when ROS Related Regulator Transcription Factor was knockout. (A) Systematic analysis of multiple GEO datasets revealed correlation between 31 well-established and 46 newly identified APC ligands in ROS related transcription factor KO GEO datasets. (B) Summary table of the 31 known inhibitory APC ligands, including the number of significantly (p < 0.05) upregulated or downregulated genes in each GEO dataset. Detailed expression profiles of the 31 known inhibitory APC ligands across the GEO datasets summarized in SFigure 6A. (C) Summary table of the 46 newly identified inhibitory APC ligands, including the number of significantly (p < 0.05) upregulated or downregulated genes in each GEO dataset. Detailed expression profiles of the 46 newly identified APC ligands across the GEO datasets summarized in SFigure 6B.
(A).
(B).
| 31 Known APC ligands Gene Expression Omnibus (GEO) | Features | Source Name | Experimental Conditions/ Group Comparison | P<=0.05 | (#)Upregulation | (#)Downregulation |
| --- | --- | --- | --- | --- | --- | --- |
| GSE75452 | Knockout of ROS-related transcription factors | Human Lung Cancer Cell Line(A549) | NFE2L2 siRNA vs Ctrl siRNA | 8 | 8 | 0 |
| GSE234443 | | Human Epitheliad Cell | Foxm1 siRNA RPTEC vs Negative Control siRNA RPTEC | 6 | 6 | 0 |
| GSE255410 | | Human Primary T Cells | CD19-FOXO1 KO vs CD19-AAVS | 5 | 2 | 3 |
| GSE120941 | | Human Multiple Myeloma Celline ME-1 | FOXO1 KO vs Control | 10 | 4 | 6 |
| GSE171572 | | Human Cell Line MCF10A | TP53(-/-) vs WT | 6 | 5 | 1 |
| GSE132327 | | Human Gastric Cancer Cell line | TP53(-/-) vs WT | 0 | 0 | 0 |
| GSE29988 | | Mouse Renal Neoplasia | Hif1a/Fh1 KO vs Control | 9 | 7 | 2 |
| GSE196129 | | Human Serum | PBRM1 shRNA vs Off Target shRNA | 6 | 1 | 5 |
| GSE102806 | | Human Clear Renal Cell Carcinoma Cell Line | PBRM1 shRNA vs Off Target shRNA | 4 | 2 | 2 |
| GSE227232 | | Human HCT116 Cells | NRF1 KD vs Control | 3 | 3 | 0 |
| GSE35124 | | Mouse Liver | NRF1 CKO vs Control | 13 | 13 | 0 |
| GSE241066 | | Human Esophageal Squamous Cell Carcinoma Cells | si-KAT8 vs si-Nc | 11 | 7 | 4 |
| GSE43940 | | Mouse Embryonic Ureter | TSHZ3 Lac2/Lac2 vs WT | 1 | 0 | 1 |
| GSE102870 | | Human IPSC-derived Hepatocytes | NR1H4 Homozygous Knockout vs WT | 5 | 5 | 0 |
| # of unique modulated Ligands | | | 22 / 31 | | | |
(C).
| 46 UnKnown APC ligands Gene Expression Omnibus (GEO) | Features | Source Name | Experimental Conditions/ Group Comparison | P<=0.05 | (#)Upregulation | (#)Downregulation |
| --- | --- | --- | --- | --- | --- | --- |
| GSE75452 | Knockout of ROS-related transcription factors | Human Lung Cancer Cell Line(A549) | NFE2L2 siRNA vs Ctrl siRNA | 14 | 8 | 6 |
| GSE234443 | | Human Epitheliad Cell | Foxm1 siRNA RPTEC vs Negative Control siRNA RPTEC | 11 | 9 | 2 |
| GSE255410 | | Human Primary T Cells | CD19-FOXO1 KO vs CD19-AAVS | 6 | 4 | 2 |
| GSE120941 | | Human Multiple Myeloma Celline ME-1 | FOXO1 KO vs Control | 7 | 2 | 5 |
| GSE171572 | | Human Cell Line MCF10A | TP53(-/-) vs WT | 10 | 9 | 1 |
| GSE132327 | | Human Gastric Cancer Cell line | TP53(-/-) vs WT | 5 | 4 | 1 |
| GSE29988 | | Mouse Renal Neoplasia | Hif1a/Fh1 KO vs Control | 16 | 13 | 3 |
| GSE196129 | | Human Serum | PBRM1 shRNA vs Off Target shRNA | 21 | 10 | 11 |
| GSE102806 | | Human Clear Renal Cell Carcinoma Cell Line | PBRM1 shRNA vs Off Target shRNA | 6 | 4 | 2 |
| GSE227232 | | Human HCT116 Cells | NRF1 KD vs Control | 5 | 5 | 0 |
| GSE35124 | | Mouse Liver | NRF1 CKO vs Control | 33 | 33 | 0 |
| GSE241066 | | Human Esophageal Squamous Cell Carcinoma Cells | si-KAT8 vs si-Nc | 27 | 14 | 13 |
| GSE43940 | | Mouse Embryonic Ureter | TSHZ3 Lac2/Lac2 vs WT | 1 | 0 | 1 |
| GSE102870 | | Human IPSC-derived Hepatocytes | NR1H4 Homozygous Knockout vs WT | 9 | 5 | 4 |
| # of unique modulated Ligands | | | 46 /46 | | | |
